# Origin of subgenomes in the circumboreal, allopolyploid, carnivorous plant *Drosera anglica*

**DOI:** 10.1002/ajb2.70170

**Published:** 2026-03-02

**Authors:** Rebekah A. Mohn, Ya Yang

**Affiliations:** ^1^ Department of Plant and Microbial Biology University of Minnesota 1445 Gortner Avenue St. Paul 55108 MN USA; ^2^ Present address: Center for Tree Science, The Morton Arboretum 4100 Illinois Route 53 Lisle 60532 IL USA

**Keywords:** boreal, Caryophyllales, Droseraceae, genome size, Hawaii, HybPhaser, phylogenomics, polyploidy, subgenome phasing, transcriptome

## Abstract

**Premise:**

The parentage of the widespread allopolyploid *Drosera anglica*, a member of the carnivorous sundew genus, remains uncertain despite over 100 years of morphological, cytological, and, more recently, molecular study.

**Methods:**

Using transcriptomic and genomic data from 12 species of *Drosera* sect. *Drosera*, including four *D. anglica* populations and a population sometimes identified as disjunct *D. intermedia*, we assembled genes in HybPiper and phased sequences in HybPhaser. We estimated species relationships with phylogenetic and pairwise genetic distance methods and ploidy with heterozygosity and flow cytometry measurements. Additionally, we expanded represented taxa by analyzing new and previously published *rbcL* sequences.

**Results:**

Sequences from phased subgenomes of *D. anglica* were highly similar to *D. rotundifolia* (99.60–99.80%) and *D. linearis* (99.79–99.95%) and showed no evidence of multiple origins despite sampling across North America, Europe, and Hawaii. Additionally, the disjunct *D. intermedia* from Idaho had been misidentified and is *D. anglica*. *S*equences from the nuclear ribosomal region and *rbcL* of *D. anglica* were nearly identical to *D. linearis* despite their chromosomes mispairing during meiosis and counter to interpretations of limited Sanger sequencing. *Drosera anglica* is intermediate between its parental lineages in leaf shape and microhabitat; however, across *D*. sect. *Drosera*, neither leaf shape nor biogeographic distribution was a reliable indicator of phylogenetic relationships.

**Conclusions:**

*Drosera anglica* arose from allopolyploidy between the *D. linearis* lineage, representing the plastid and dominant ribosomal donor, and the *D. rotundifolia* lineage. Our study demonstrates the importance of taxon sampling and careful examination of complex phylogenomic data and presents an exemplar of analyzing allopolyploid relationships.

Allopolyploidy, a driving force of evolutionary innovation, brings together genes, regulatory elements, and variants in ways that differ from either parent (Nieto Feliner et al., 2020). As the allopolyploid genome undergoes subsequent restructuring, gene copies may be lost, exposed to new regulatory elements, or located in different epigenetic contexts (Nieto Feliner et al., 2020). Identifying the parental lineages is necessary to understand the long‐term trajectory of these changes and their impact on species diversification. A combination of careful morphological, cytological, and sequence approaches, complemented by distribution and habitat information, is ideal, as each line of evidence on its own can be insufficient or even misleading.

*Drosera* L. (Droseraceae Salisb., Caryophyllales) is a carnivorous plant genus of ~250 species worldwide. While most species in *Drosera* are restricted in distribution, *D. anglica* Huds. (English sundew) is one of the most widespread and is found in boreal North America, Europe, and Asia and in Hawaii (Lowrie et al., 2017; Fleischmann et al., 2018). Previous molecular phylogenetic studies based on one to five Sanger‐sequencing markers have placed *D. anglica* in *D*. subg*. Drosera*, sect. *Drosera* (Rivadavia et al., 2003; Veleba et al., 2017; Fleischmann et al., 2018). Based on morphological and cytological evidence, *D*. sect. *Drosera* includes 28 species worldwide (Fleischmann et al., 2018). Of them, only three species have distributions in the boreal zone: Both *D. anglica* and *D. rotundifolia* L. are circumboreal with disjunct populations in tropical mountains, and the third species, *Drosera linearis*, is restricted to boreal North America (Figure 1).

**Figure 1:**
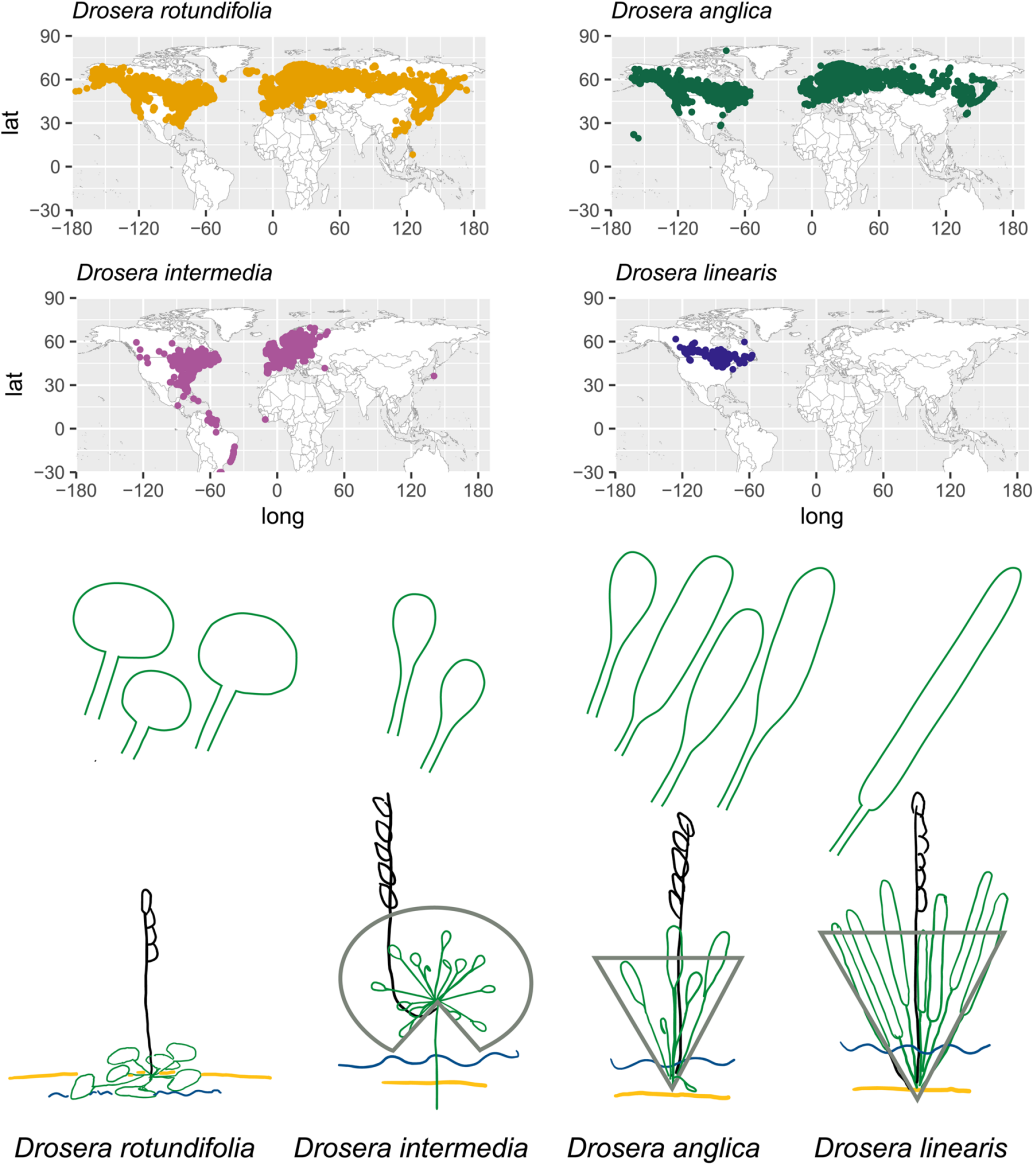
Leaf shape, angle of the petiole, and shape of the peduncle among *Drosera anglica*, its parental lineages: *D. rotundifolia* and *D. linearis*. *Drosera intermedia* is included for comparison because it is often confused with *D. anglica*. The yellow line represents the substrate, the blue line represents the water level, and the gray outline depicts the distribution of leaves as erect or radiating. lat: latitude; long: longitude.

Most species in *Drosera* sect. *Drosera* have a diploid chromosome number of 2*n* = 20, except *D. anglica* (2*n* = 40), some populations of *D. spatulata* Labill. (2*n* = 20, 40 presumably autopolyploid), *D. tokaiensis* (Komiya & C. Shibata) T. Nakam. & K. Ueda (2*n* = 60, an allopolyploid with genomes of *D. spatulata* and *D. rotundifolia* in Japan; Nakamura and Ueda, 1991; Hoshi et al., 2010), and *D. neocaledonica* Raym.‐Hamet (2*n* = 60, a species restricted to New Caledonia; Kondo and Segawa, 1988; Gervais and Gauthier, 1999; Mohn et al., 2023). The widely distributed *D. anglica*, however, is known to be of hybrid origin, with uncertain parental lineages, despite a long history of cytological and molecular studies (Table 1). The observation that hybrids between *D. rotundifolia* (2*n* = 20) and *D. anglica* (2*n* = 40) resulted in 10 bivalent (II) and 10 univalent (I) chromosome pairs (from this point referred to as 10II, 10I; Table 1) led to the hypothesis that *D. anglica* was of allopolyploidy origin between *D. rotundifolia* and another species (Rosenberg, 1903, 1904, 1909; Winge, 1917). The second parental lineages were thought to be either *D. intermedia* Hayne or *D. linearis* Goldie, given their overlapping distributions and similarities in habitat and morphology compared to *D. anglica* (Figure 1; Wood, 1955; Gervais and Gauthier, 1999).

**Table 1:** Published observations of chromosome pairing from naturally occurring hybrids or crossing experiments between *Drosera anglica* and species in *D*. sect. *Drosera*. Chromosomes have been observed to form univalent (I), bivalent (II), trivalent (III), and quadrivalent (IV) pairs.

Species	Distribution	Chromosome count	Meiotic pairing when hybridized with *D. anglica*	Reference
***D. filiformis***	East Coast North America	2*n* = 20	12‐13,17 I; 5‐7 II; 1‐2 III	Kondo and Segawa (1988)
***D. linearis***	Boreal North America	2*n* = 20	9‐13 I; 7‐10 II; 0‐1 III	Gervais and Gauthier (1999)
***D. anglica***	Circumboreal, Hawaii	2*n* = 40	20 II	Kondo and Segawa (1988)
***D. intermedia***	Eastern North America, South America, Europe	2*n* = 20	10 I; 10 II	Kondo and Segawa (1988)
***D. capillaris***	Eastern North America, South America	2*n* = 20	13‐14 I; 7‐8 II; 0‐1 III	Kondo and Segawa (1988)
***D. rotundifolia***	Circumboreal, Southeast Asia	2*n* = 20	10 I; 10 II	Kondo and Segawa (1988)
***D. spatulata***	Eastern Oceania, Southeast Asia	2*n* = 40	4,6,10 I; 10, 12, 14 II; 1‐4 IV	Kondo and Segawa (1988)
***D. tokaiensis***	Southeast Asia	2*n* = 60	10, 12, 16 I; 17, 19, 20 II	Kondo and Segawa (1988)

*Drosera anglica* and *D. intermedia* have very similar leaf shapes and can be easily confused, especially in herbarium specimens (Figure 1). The plants, especially early in the vegetative stage, have few distinguishing features. *Drosera intermedia* occurs in eastern North America, Europe, and South America. It has been reported from two disjunct populations in northern Idaho and southern Idaho, more than 1000 km from the nearest populations (Mellichamp, 2016; Lowrie et al., 2017). The Idaho populations are state‐listed as a rare plant, but some botanists have suggested that they may be misidentified *D. anglica* populations (Lowrie et al., 2017; Rice, 2019). While *D. rotundifolia* and *D. intermedia* rarely form hybrids with each other in the wild (Grima, 2020), *D. anglica* and *D. intermedia* form 10 II and 10 I pairs when hybridized artificially (Table 1; Kondo and Segawa, 1988). Work evaluating the isozymes (proteins from different gene copies) of European *Drosera* found that *D. anglica* and *D. rotundifolia* shared allozymes (proteins from the same gene copy) and *D. intermedia* and *D. anglica* did not, contradicting the evidence from morphology and chromosome pairing (Seeholzer, 1993).

Alternatively, *D. linearis* has been hypothesized as the other parental lineage of *D. anglica* based partly on the similarity of their microhabitats (Figure 1). *Drosera anglica* occurs in fen strings (ridges in patterned fens), fen edges, and wetter regions of bogs. This microhabitat is intermediate between the often calcium‐rich fen flark (a depression in a patterned fen) microhabitat of *D. linearis* and the drier, more acidic sphagnum hummock microhabitat of *D. rotundifolia. Drosera anglica* is also intermediate in leaf shape between *D. linearis* and *D. rotundifolia* (Wood, 1955). However, the chromosomes of *D. linearis* do not pair properly with those of *D. anglica* (Table 1). When hybridized in the wild, *D. anglica* × *D. linearis* forms 9‐13 I, 7‐10 II, and 1 III during meiosis (Gervais and Gauthier, 1999). Chromosomes in wild hybrids between *D. rotundifolia* and *D. linearis* do not pair properly either (2‐10 I, 3‐7 II, and 1‐3 III; Wood, 1955). Although fertile neoallopolyploid hybrids of *D. rotundifolia* and *D. linearis* have been found in nature, they are slightly different in appearance compared to *D. anglica* (Wood, 1955). Hybrids of *D. anglica* with *D. spatulata*, *D. tokaiensis*, *D. capillaris* Poir., and *D. filiformis* Raf. also produce mismatched chromosomes (Table 1; Kondo and Segawa, 1988; Gervais and Gauthier, 1999).

Previous molecular phylogenetic work on *Drosera* has been inadequate to clarify *D. anglica*'s parentage. Rivadavia et al. (2003) sampled 10 species of *Drosera* sect. *Drosera* across its range of distribution but did not include *D. intermedia* or *D. linearis*. They found that the plastid RUBISCO large subunit (*rbcL*) nucleotide sequences of *D. anglica* and *D. rotundifolia* differed by only 3 bp and hypothesized that *D. rotundifolia* represents the plastid donor lineage (Rivadavia et al., 2003). However, this and other more recent phylogenetic analyses of *Drosera* have lacked phylogenetic signal for resolving the relationships within the recently diversified *D*. sect. *Drosera* (Rivadavia et al., 2003; Biswal et al., 2017; Veleba et al., 2017; Mohn et al., 2023). Additionally, most studies have lacked the necessary sampling due to not including *D. linearis*, one of the candidate parental species (Rivadavia et al., 2003; Biswal et al., 2017; Veleba et al., 2017).

Increased sampling of nuclear loci is needed to detect both parental lineages of *D. anglica* in the context of the recently diverged *D*. sect. *Drosera*. Transcriptomes provide thousands of loci, each with relatively long sequences informative for evaluating discordance among taxa with short branch lengths. It is also an effective genome subsampling approach to tease apart subgenomes. Additionally, transcriptome data sets can be readily reused and combined with genome resequencing and target enrichment data sets to address questions related to phylogenetic and molecular evolution.

With this information in mind, we sequenced transcriptomes from one to four populations for each of 12 species from across *D*. sect. *Drosera*, with a special emphasis on the three boreal species and species that have previously been hypothesized to be involved in the parentage of *D. anglica*. We reconstructed gene trees and species trees, phased subgenomes, and analyzed genetic distance and allele diversity to answer the following questions: (1) What are the parental lineages of *Drosera anglica*, and how does that compare to previous cytological findings? (2) Is there any evidence for multiple independent or ongoing allopolyploidy origins of *D. anglica*? (3) Is the northern Idaho population of “*D. intermedia*” actually *D. intermedia* or *D. anglica*?

## MATERIALS AND METHODS

### Sampling and collection

To maximize the chance that our sampling included the parents of *D. anglica*, we sampled species from across *D*. sect. *Drosera*, with an emphasis on putative parents of *D. anglica*. We selected species based on the previously published *rbcL* phylogeny (Rivadavia et al., 2003) representing major clades, different geographic distributions, and diverse leaf morphologies. For *D. anglica*, we sampled across its distribution and included multiple populations from its hypothesized parents.

We sampled one European, one Hawaiian, and two North American populations of *D. anglica*; two populations each of *D. linearis*, *D. rotundifolia*, *D. capillaris*, and one population for three of the four remaining North American species and four South American species. We also sampled the northern “*D. intermedia*” Idaho population, whose identification has been disputed in the recent literature. In addition, we downloaded a publicly available transcriptome from a Russian population of *D. rotundifolia* (NCBI SRA: SRR8948654; Gruzdev et al., 2019). In total, we sampled seven of the eight species that occur in North America (with the unsampled species representing a closely related taxon that is sometimes treated as a subspecies of *D. filiformis*), seven of 18 that occur in South American, four of the six species that occur in Asian/Europe, and two of the three species that occur in Oceania (Lowrie et al., 2017; Fleischmann and Gonella, 2018). This sampling included lineages from across *D*. sect. *Drosera* and lineages proximal to *D. anglica* (Rivadavia et al., 2003; Mohn et al., 2023). For clarity, we will refer to each sample by the species name, and for species with multiple collections, the species name is followed by the location abbreviation in parentheses. To root the tree, we used the published *D. spatulata* reference genome (Palfalvi et al., 2020) since it is diploid and sister to the rest of *D*. sect. *Drosera* (Veleba et al., 2017). For the outgroup, we used *D. prolifera* C.T. White, a diploid in *D*. subg. *Drosera* but outside of *D*. sect. *Drosera* (Veleba et al., 2017; Fleischmann et al., 2018).

Newly generated transcriptomes came from field‐collected and cultivated samples. For most North American samples, we collected the whole plant with roots and some substrate in the field and placed it in a zip‐locking bag. When we returned to the trailhead (within 3 h of collection), we transferred the aboveground portion of the plant into an 8‐mL Nalgene bottle (Thermo Fisher Scientific, Waltham, MA, USA) and froze the sealed bottle in liquid nitrogen. Cultivated plants were sourced from a reputable specialty grower, Best Carnivorous Plants (Ostrava‐Poruba, Czechia), for the South American and European populations (Table 2; Appendix S1: Table S1; photo vouchers: Appendix S2). Tissue from cultivated plants was collected, immediately placed in a 2‐mL lysing tube with Lysing Matrix A (MP Biomedicals, Irvine, CA, USA), and flash frozen in liquid nitrogen. To avoid contamination while collecting the samples, we wore gloves and cleaned tweezers between samples with 70% v/v ethanol, then RNase*Zap* (Invitrogen, Carlsbad, CA, USA). All samples were ground using the FastPrep‐24 5 G bead beating grinder (MP Biomedicals) and Lysing Matrix A in dry ice with the CoolPrep adapter (MP Biomedicals). RNA was extracted using the PureLink Plant RNA Reagent (Invitrogen; see Appendix S3 for modifications to the manufacturer's protocols; Yang et al., 2017). Library preparation and sequencing were done by either the University of Minnesota Genomics Center (UMGC, St. Paul, MN, USA; seven samples) or Novogene, (Sacramento, CA, USA; 11 samples; Appendix S1: Table S2). At UMGC, given the relatively low RNA integrity number (RIN), the libraries were prepared using Illumina Ribo‐Zero Plus rRNA Depletion Kit (San Diego, CA, USA) to avoid 5′ bias with the more standard poly(A) enrichment. Libraries were sequenced with either 125‐bp paired‐end reads on the Illumina HiSeq. 2500 or 150‐bp single‐end reads on the NextSeq. 550 (Appendix S1: Table S2). We requested paired‐end reads, but the sequencing facility accidentally performed single‐end read library prep, and the remaining samples were discarded. Library preparation at Novogene used the NEBNext Ultra II Directional RNA Library Prep kit (New England Biolabs, Ipswich, MA, USA) followed by sequencing with 150 paired‐end reads on the Illumina NovaSeq. 6000 platform (Appendix S1: Table S2).

**Table 2:** Sample source, voucher information, and genome sizes. Locality is the United States unless otherwise specified. Published genome size estimates from an unspecified source population are reported in separate rows with footnotes. Genome size standards for newly published genome sizes are in Appendix S1: Table S1.

Sample	Source (Sample location abbreviation)	Collection number (Voucher)	Diploid genome size (Mb)
*D. anglica* (CZ)	(cult.) Best Carnivorous Plants (BCP ID# D1594, D1614). Locality: Sumava Mts, Southern Bohemia, Czech Republic (CZ)	RM298A (Appendix S2: Figure S1: Photo)	—
*D. anglica* (MN)	Lost River Peatlands Scientific Natural Area (SNA), Minnesota (MN)	RM230 (MIN)	—
*D. anglica* (HI)	Wahiawa Bog, Kauaʻi, Hawaii (HI)	DNA accession: CM635	—
		Collection: Motley 1440; Population voucher: B.C. Stone 3735 (BISH)	
*D. anglica*	Population unspecified (Veleba et al., 2017)		4715^a^
*D. anglica* (WA)	Priest Lake Ranger District, Washington (WA)	RM217 (MIN)	4640
*D. brevifolia*	Cherry Orchard Natural Area Preserve, Virginia	RM211 (MIN)	1636 (Mohn et al., 2023)
*D. capillaris* (FL)	(cult.) Best Carnivorous Plants (BCP ID# D1092). Locality: Florida Panhandle (FL)	RM240 (Appendix S2: Figure S2: Photo)	—
*D. capillaris* (VA)	Cherry Orchard Natural Area Preserve, Virginia (VA)	RM210 (MIN)	—
*D. esmeraldae*	(cult.) Best Carnivorous Plants (BCP ID# D761). Locality: Cerro Duida, Venezuela	RM241 (Appendix S2: Figure S3: Photo)	—
*D. felix*	(cult.) Best Carnivorous Plants (BCP ID# D684, D923, D1834). Locality: Tuku Muruku, Gran Sabana, Brazil	RM245 (MIN; Appendix S2: Figure S4: Photo)	2198 (Mohn et al., 2023)
*D. filiformis*	Webb's Mill Bog, New Jersey (NJ)	RM208 (MIN)	—
*D. filiformis*	Population unspecified (Veleba et al., 2017)	—	4877^a^
“*D. intermedia*” (ID)	Bonner's Ferry Ranger District, Idaho (ID)	RM218 (MIN)	5303
*D. intermedia* (NJ)	Webb's Mill Bog, New Jersey (NJ)	RM207 (MIN)	—
*D. intermedia*	Population unspecified (Veleba et al., 2017)	—	2516^a^
*D. linearis* (MN)	Lost River Peatlands SNA, Minnesota (MN)	RM228 (MIN)	—
*D. linearis* (MT)	Lincoln Ranger District, Montana (MT)	RM219 (MIN)	—
*D. prolifera*	(cult.) Best Carnivorous Plants (BCP ID# D953, D1596, D1783). Locality: Unknown.	RM294 (MIN)	—
*D. prolifera*	Population unspecified (Veleba et al., 2017)	—	502^a^
*D. roraimae*	(cult.) Best Carnivorous Plants (BCP ID# D1026). Locality: Summit of Mt. Roraima (which borders Brazil, Guyana, Venezuela).	RM242 (Appendix S2: Figure S5: Photo)	—
*D. roraimae*	Population unspecified (Veleba et al., 2017)	—	2683^a^
*D. rotundifolia* (ID)	Priest Lake Ranger District, Idaho (ID)	RM214 (MIN)	1933
*D. rotundifolia* (NJ)	Webb's Mill Bog, New Jersey (NJ)	RM209 (MIN)	—
*D. rotundifolia* (RUS)	Russia: Moscow region, wetland (RUS) SRR8948654	—	—
*D. rotundifolia*	Population unspecified (Veleba et al., 2017)	—	2331^a^
*D. solaris*	(cult.) Best Carnivorous Plants (BCP ID# D982). Locality: Mt Yakontipu, Pakaraima Mountains, Guyana	RM237 (MIN)	2429 (Mohn et al., 2023)
*D. spatulata*	Palfalvi et al., 2020	—	646^b^

^a^ Species genome sizes from unspecified population (Veleba et al., 2017).

^b^ Genome size from unspecified population (Palfalvi et al., 2020).

For a Hawaiian population of *D. anglica*, we obtained DNA from the Hawaiian Plant DNA Library (Morden et al., 1996; Randell and Morden, 1999). This sample was sequenced by Novogene with the ABclonal DNA Library Prep Kit (ABclonal, Woburn, MA, USA), 350‐bp size selection after fragmentation, and 150‐bp paired‐end reads on the Illumina NovaSeq. 6000 platform.

### Read processing and sequence assembly

For the transcriptomic data, we used the previously published pipeline https://bitbucket.org/yanglab/phylogenomic_dataset_construction/ (Yang and Smith, 2014; Morales‐Briones et al., 2021). The pipeline started with the Python script filter_fq.py to remove sequencing errors using Rcorrector with default settings (Song and Florea, 2015) and to remove adapters and trim low‐quality base pairs using Trimmomatic (Bolger et al., 2014; ILLUMINACLIP:TruSeq_adapters.fa:2:30:10 SLIDINGWINDOW:4:5 LEADING:5 TRAILING:5 MINLEN:25). It then used Bowtie2 (Langmead and Salzberg, 2012) to map and separate organellar reads and FastQC (Andrew, 2010) to detect and filter overrepresented reads. The remaining transcriptome reads were assembled de novo with Trinity version 2.5.1 (Haas et al., 2013).

Croco version 1.1 (Simion et al., 2018) was used to test for cross‐contamination. The cleaned reads and the assembled transcripts were fed into Croco in two groups: one from paired‐end and the other from single‐end data sets because Croco can process only one read configuration per run.

Visual inspection of reads mapped to initial assemblies indicated that the reads from the single‐end sequencing batch (*D. rotundifolia* [ID], *D. anglica* [WA], and “*D. intermedia*” [ID]) often had a T at the 3′ end, likely as part of the adapter sequence. Therefore, one base was removed from the 3′ end of these three samples for subsequent analyses unless otherwise stated.

For the whole‐genome resequencing data, we also filtered organellar reads for separate processing. To reduce the data to only relevant reads, we used BBmap allowing 10 substitutions per read and 80% identity to map reads to consensus assemblies of *D. rotundifolia* (NJ) and *D. linearis* (MN) generated by HybPhaser (see below).

### Synthetic in silico hybrids

As a positive control to evaluate our ability to tease apart subgenomes in allopolyploids, after cleaning the raw reads, we combined 6,666,667 150‐bp paired‐end (PE) reads from *D. linearis* (MT) and 8,000,000 125‐bp PE reads from *D. rotundifolia* (NJ) to make a synthetic in silico hybrid that roughly resembled *D. anglica* in read coverage. This difference in the number of reads made up for the difference in read length between 125‐bp PE reads of *D. rotundifolia* (NJ) and 150‐bp PE reads of *D. linearis* (MT). Since single‐end reads may suffer from additional challenges in phasing, we generated a second synthetic hybrid with the same reads but without pairing information. From now on, these two data sets will be referred to as synthetic hybrids.

### Distribution of synonymous distance (Ks) estimated using unfiltered and de novo assembled transcripts

The divergence of subgenomes within *D. anglica* was visualized with Ks plots. To do so, all assembled and unfiltered transcripts from Trinity (before the 3′ end of *D. rotundifolia* [ID], *D. anglica* [WA], and “*D. intermedia*” [ID] was trimmed) were translated with TransDecoder version 5.3.0 (Haas, 2015). Within‐species Ks plots were calculated as described by Yang et al. (2018). We removed Ks values < 0.01 before visualizing Ks distributions because heterozygosity and isoforms often contribute to Ks values less than 0.01.

### Selecting target genes for targeted assembly using HybPiper

We chose targeted assembly for phylogenetic analyses given the tools available for phasing subgenomes and the ability to combine our genome and transcriptome data sets. To select target genes that are single‐copy and well supported by transcriptome data and to reduce computational time, we did an initial round of phylogenomic analysis with transcriptome assemblies from two *D. linearis*, two *D. rotundifolia* (before the 3′ end of *D. rotundifolia* [ID] was trimmed), *D. intermedia* (NJ), and the CDS file from the *D. spatulata* genome annotation (Palfalvi et al., 2020). This was done following the Yang and Smith pipeline (Yang and Smith, 2014; Morales‐Briones et al., 2021).

Using the Trinity transcriptome assemblies and TransRate version 1.0.3 (Smith‐Unna et al., 2016), we removed transcripts with nucleotides of mapped reads poorly matching the assembled transcript [TransRate contig (*C*) component score *s*(*C*_nuc_) ≤ 0.25], low read coverage [*s*(*C*_cov_) ≤ 0.25], or paired‐end reads misaligned [*s*(*C*_ord_) ≤ 0.5] in paired‐end read samples. Chimeric transcripts with multiple open reading frames stitched together in opposite directions, each with at least 100 bp of query length with at least 30% similarity in BLASTx hits against *Beta vulgaris* L. proteins were removed (Yang and Smith, 2013). The resulting transcripts were translated with TransDecoder with *Arabidopsis thaliana* (L.) Heynh. and *Beta vulgaris* reference proteomes. Finally, the CDS were further reduced with CD‐HIT‐EST (Li and Godzik, 2006) to remove sequences with >99% similarity using a 10‐bp word length.

To cluster the resulting CDS files, hits from an all‐by‐all BLASTn (Altschul et al., 1990; Camacho et al., 2009) search with at least a 30% length coverage of both sequences were input into mcl (Van Dongen, 2008) with an inflation value of 1.4. The resulting clusters were each aligned with MAFFT version 7.475 (Katoh and Standley, 2013) using the generalized affine gap cost for pairwise alignments with 1000 iterations. Alignments were then trimmed with Phyx (Brown et al., 2017), removing columns with >90% missing data, and gene trees were estimated using RAxML (version 8.2.11). Using TreeShrink (Mai and Mirarab, 2018), we trimmed spurious tips that were in the 0.4 quantile. Then we removed monophyletic tips of the same sample, leaving the one with the highest number of aligned characters in the trimmed alignment. We then visually inspected the resulting gene trees. We also cut internal branches that were longer than 0.1 substitutions per site because internal branches among our sampled species were mostly <0.06 and branches longer than 0.1 were due to deeper paralogs or spurious sequences. We retained trees with all six taxa and re‐aligned the sequences with MAFFT. Terminal branches 10× longer than their sister or more than 1.0 substitutions per site were trimmed. We thinned monophyletic and paraphyletic tips from the same sample to the one with the highest number of aligned characters in the trimmed alignment. Finally, we selected one‐to‐one orthologs present in all six samples and used the *D. spatulata* coding sequences for resulting 6443 genes as target sequences in downstream analyses

### Targeted assembly with HybPiper

Using HybPiper 2.0 (Johnson et al., 2016) with default settings unless specified, we first mapped reads to the 6443 *D. spatulata* target genes using bwa (Li, 2013) and assembled the mapped reads into transcripts with SPAdes (Bankevich et al., 2012). We required a read depth ≥4, given the uneven read coverage of transcriptome data. For the transcriptomes, we used the setting ‐‐no_stitched_contig to minimize the risk of chimeric sequences. Assembled transcripts for each gene were compared to each other and the reference using Exonerate version 2.2.0 (Slater and Birney, 2005) and were retained if the hits had ≥85% identity.

### Reference‐based phasing with HybPhaser

We used the HybPhaser pipeline to identify polyploids, identify parental lineages, and phase the subgenomes (Nauheimer et al., 2021). HybPhaser consists of four steps: visualizing the distribution of single‐nucleotide polymorphisms in each sample to detect potential hybrids and polyploids, determining whether samples belong to multiple clades by mapping to clade references, phasing reads to the appropriate clade references, and then re‐assembling the phased reads in HybPiper. First, loci with less than 20% of samples or that covered <10% of the length of the target sequence were removed. Using only the reads mapped to the target by bwa in HybPiper, HybPhaser version 2.1 (Nauheimer et al., 2021) generated a consensus sequence for each gene in each sample. For a second allele to be called at a site according to the default settings, there must be a read depth of at least 10 at the site, and the second allele must be supported by at least four reads and 15% of the reads. Allele divergence, the percentage of SNPs per gene length, and locus heterozygosity, the percentage of loci with SNPs, were calculated by HybPhaser. In single‐copy genes in diploid species, allele divergence equals the nucleotide diversity per site (*π*). In genes with multiple closely related copies, especially in polyploid species, paralogs and homeologs also contribute to allele divergence. We identified samples with elevated heterozygosity and sequence divergence as evidence of polyploidy and/or hybrids and candidates for subgenome phasing. To select which data set to use as clade references to phase to, we gathered the genes from the initial HybPiper run and estimated gene trees with the default settings of the script fasta_to_tree_pxclsq.py (Yang and Smith, 2014; Morales‐Briones et al., 2021) using MAFFT v7.475 (Katoh and Standley, 2013), Phyx version 1.01 (Brown et al., 2017), and RAxML version 8.2.11 (Stamatakis, 2014). We then estimated the species tree in ASTRAL version 5.7.8 (Zhang et al., 2017). We selected putative diploid references with low heterozygosity and sequence divergence that represented different clades within *D*. sect. *Drosera*. Each sample was then mapped to the consensus assemblies of the clade references. Samples that had both high heterozygosity and sequence divergence and mapped to multiple clade references at approximately equal rates were then phased. To phase subgenomes, we used HybPhaser to map reads from a sample to consensus assemblies from both references using BBMap version 38.96 (Bushnell, 2014). If the reads mapped unambiguously to one of the references, the reads were sorted to the file corresponding to that reference. If they mapped to both equally, they went to both files. If they did not map to either reference, they were removed. The resulting two files of phased reads were each assembled to the original 6443 *D. spatulata* genes using HybPiper.

The resulting phased homeologs from HybPhaser and unphased genes from the remaining samples were again aligned using MACSE_OMM v. 12.01 with default settings, which takes codon sequence into account (Ranwez et al., 2011, 2021), and alignments trimmed using Phyx (Brown et al., 2017), requiring a minimum of 5% column occupancy. A subset of resulting alignments was visually inspected to ensure proper assembly and phasing. Using raxml_bs_wrapper.py (Yang and Smith, 2014; Morales‐Briones et al., 2021), we estimated gene trees with the GTRCAT substitution model and 100 bootstrap replicates in RAxML. Genes with only one copy per sample or subgenome were rooted with *D*. *prolifera* using pxrr in Phyx (Brown et al., 2017). ASTRAL was then used to estimate the species tree from the gene trees. Gene tree discordance was then calculated by PhyParts (Smith et al., 2015), requiring a minimum local bootstrap of 50. Trimmed alignments with at least 50 bp in aligned length were concatenated for phylogenetic reconstruction using RAxML.

### Pairwise genetic distance

In addition to tree‐based methods, we calculated the pairwise genetic distance between samples. A distance‐based method is especially informative when relationships among samples are not strictly tree‐like and when samples are very closely related. We removed MACSE‐OMM alignments with fewer than 98.0% pairwise identical base pairs (including gap‐to‐base comparisons), which removed alignments with large segments of ambiguous characters or high levels of missing data. After visually inspecting the remaining alignments, we removed alignments with ambiguous characters, removed columns with any gaps, and kept only alignments longer than 1000 bp to ensure sufficient signal. We then used bio3d version 2.4‐4 (Grant et al., 2006) in R version 4.2.3 (R Core Team, 2023) to calculate the pairwise genetic distance within each alignment. We then calculated the mean and median genetic distance between each pair of samples or subgenomes.

### *rbcL* and ribosomal sequence analysis to compare with previous Sanger‐sequencing studies

To compare our results with a broader taxon sampling based on previous Sanger sequencing data and to infer the plastid donor lineage, we assembled the plastid *rbcL* sequence from all samples. To ensure that SNPs were not due to low‐quality bases, we first performed a second, more stringent round of Trimmomatic trimming with settings LEADING:28 TRAILING:28 SLIDINGWINDOW:8:28 SLIDINGWINDOW:1:10 MINLEN:65. We then used Bowtie2 to map the reads to a reference *rbcL* sequence from *D. rotundifolia* (AB29809.1 from NCBI GenBank). We used the Bowtie2 ‐‐end‐to‐end setting and, additionally, the ‐‐no‐mixed setting for paired‐end samples. We extracted mapped reads using SAMtools v. 1.21 (Danecek et al., 2021) and BEDtools v. 2.29.2 (Quinlan and Hall, 2010) and assembled the reads from each sample using SPAdes v. 4.0.0 (Bankevich et al., 2012) with default settings. Assembled scaffolds greater than 300 bp were combined with *rbcL* sequences from Mohn et al. (2023). Each gene was aligned with PRANK v. 170427 (Löytynoja, 2014) and inspected. The direction of sequences was fixed in Geneious Prime v. 2023.0.1 (Biomatters, Boston, MA, USA) when needed, and all sequences were realigned with PRANK. A phylogenetic tree was estimated in RAxML, and sequences with spurious long branches were trimmed. The remaining sequences were realigned with PRANK, and a phylogenetic tree was estimated with RAxML and visualized with the plot.phylo command in R package ape (v. 5.7‐1; Paradis and Schliep, 2019). Due to the high efficiency of poly(A) enrichment in samples sequenced at Novogene, we were unable to recover the whole plastome for these samples.

To extract sequences from the nuclear ribosomal region, we used Bowtie2 to map the trimmed reads to a sequence of the entire ribosomal gene cistron (rDNA) of *D. rotundifolia* that included the rRNA small subunit gene, ITS1, 5.8S rRNA gene, ITS2, and the rRNA large subunit gene (NCBI GenBank accession MT784099.1). We attempted to follow the same procedures as for the *rbcL* sequences, but the resulting assemblies were fragmented due to the high copy numbers and the secondary structure of the ITS region (Biswal et al., 2017). Instead, we called variants using BCFtools mpileup and BCFtools call with default settings, except that the max depth was increased to 30,000 (Danecek et al., 2021). The aligned variable sites were extracted from the resulting VCF file using vcf2phylip.py (Ortiz, 2019) with no minimum number of samples. A phylogenetic tree was estimated from the aligned variable sites in RAxML with the GTRCAT model.

### Species distribution maps

We downloaded “Human observation” and “Preserved specimen” data from the Global Biodiversity Information Facility (GBIF) with an occurrence status of “present” for *D. anglica*, *D. intermedia*, *D. linearis*, and *D. rotundifolia* (GBIF.org, 2025). Observations were removed when they had an issue that included the words “CORDINATE_MISMATCH” or “COUNTRY_DERIVED_FROM_COORDINATES”, had missing coordinates or coordinates (0,0), or had an establishment means of “introduced” or “uncertain”. We also removed a single observation from New Zealand that was likely due to misidentification or an escape from cultivation. Given the high data density in some regions and multiple observations at some sites, we thinned the data to one point every hundredth of a degree of latitude and longitude. We then created a distribution map for each species using “world” map data from the R package maps (v. 3.4.1; Deckmyn, 2003), and R packages mapdata (v. 2.3.1; Becker and Brownrigg, 2022), ggplot2 (v. 3.4.2; Wickham, 2016), and ggpubr (v. 0.6.0; Kassambara, 2023) for visualization.

### Genome size estimation

Fresh leaf samples of *D. rotundifolia* (ID), “*D. intermedia*” (ID), and *D. anglica* (WA) were collected from the field and mailed to the Flow Cytometry Core Lab at the Benaroya Research Institute (Seattle, WA, USA) for genome size estimation. Samples were stained with a propidium iodide (PI), MgSO_4_, dithiothreitol, and Triton X‐100 buffer and analyzed with a FACScalibur flow cytometer (Becton‐Dickinson, San Jose, CA, USA) as described by Arumuganathan and Earle (1991a, 1991b). For each sample, four flow cytometry measurements were taken using an internal size standard (see Appendix S1: Table S1) and averaged for subsequent analyses. The source, voucher, and size standards used for generating new flow cytometry data are listed in Table S1 of Appendix S1.

## RESULTS

### Sampling, sequencing, assembly, and initial quality check

Of the 28 species in *D*. sect. *Drosera* (Fleischmann et al., 2018), we sampled 12 species in 20 populations. Of these 20 samples, 17 were newly generated transcriptome data sets, and one, *D. anglica* (HI), was a newly generated whole‐genome shotgun sequencing data set (Appendix S1: Table S2). The RNA integrity number (RIN) from the successfully sequenced RNA samples ranged from 2.0 to 7.3. For each data set, raw sequencing reads ranged from 22 million to 26 million single‐end reads or 20 to 44 million paired‐end reads (Appendix S1: Table S2). We recovered the fewest genes in HybPiper for the samples with the fewest reads after read quality and plastid read filtering. This corresponded to one of the two batches of sequencing libraries prepared with Ribozero, in which the plastid reads accounted for a significant portion of the reads.

Croco found no evidence of cross‐contamination among paired‐end samples. For the three single‐end data sets, Croco flagged 2–13% of assembled transcripts from each. However, these three, *D. rotundifolia* (ID), *D. anglica* (WA), and “*D. intermedia*” (ID), are too genetically similar to distinguish cross‐contamination from identical short reads. Therefore, we proceeded using all the samples.

All transcriptomes shared a Ks peak at around 1.8 and a second, more recent Ks peak at around 0.7, corresponding to the shared whole‐genome duplication events early in eudicots and early in Droseraceae, respectively (Walker et al., 2017; Palfalvi et al., 2020). No additional, more recent Ks peaks were distinguishable from isoforms and noise in any of the samples (Appendix S4).

### Few *D. anglica* paralogs were detected by HybPiper despite high nucleotide diversity

Gene clustering and tree‐based ortholog inference using *D. spatulata* CDS from genome annotation and transcripts assembled de novo from *D. intermedia*, *D. rotundifolia*, and *D. linearis* resulted in 6443 genes with one copy per sample to be used as targets for HybPiper assembly. Of the 6443 target genes, 4497 to 6393 were assembled in each sample. Of these, HybPiper raised long paralog warnings for 92–841 genes in each data set that had multiple assembled transcripts that each covered >50% of the target, and 112–2311 genes for paralogs with insufficient read coverage (<4) to assemble to at least 50% the length of the reference (low coverage from here on). Paired‐end *D. anglica* samples and the paired‐end synthetic hybrid had a higher number of long (501–841 vs. <229) and low‐coverage (1805–2311 vs. <500) paralogs compared to other paired‐end samples. In the *D. anglica* sample with single‐end reads, only 320–332 long and 456–458 low‐coverage paralogs were detected. The synthetic hybrids had only 493–557 long and 848–921 low‐coverage paralogs, indicating that many of the homeologs were assembled into a single chimeric copy.

**Figure 2:**
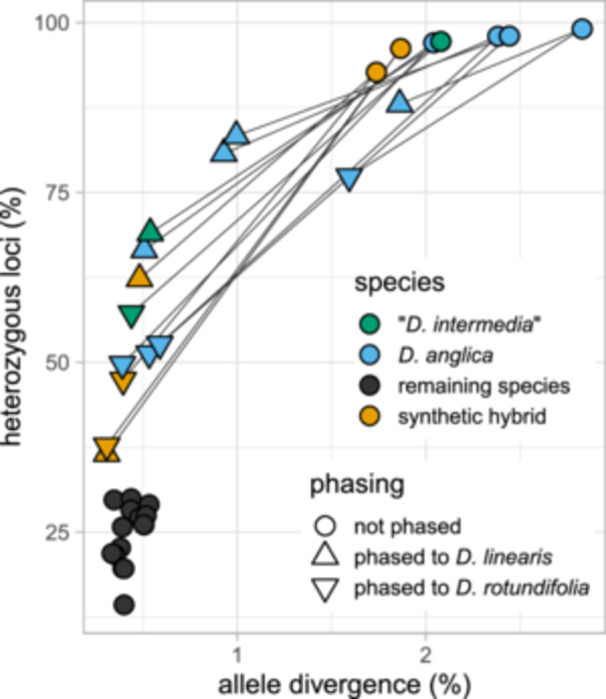
*Drosera anglica* and “*D. intermedia*” (ID) samples had much higher allele divergence and locus heterozygosity than the remaining species in *D*. sect. *Drosera*, at levels similar to the synthetic hybrid. After phasing, the allele divergence and locus heterozygosity of each sample decreased but remained higher than those of the diploid species. Lines connect phased vs. unphased datasets from the same sample. Values for allele divergence (%) and heterozygous loci (%) are in Appendix S1: Table S4.

Visualizing SNPs as the initial steps of the HybPhaser pipeline found that all *Drosera anglica* samples, “*D. intermedia*”, and the synthetic hybrids had much higher locus heterozygosity and allele divergence than diploid samples of *D*. sect. *Drosera*. Locus heterozygosity, the percentage of genes with SNPs, ranged from 97% to 99% in *Drosera anglica* and “*D. intermedia*” (ID), while in all other samples ranged from 14% to 30% (Figure 2; Appendix S1: Table S4). Similarly, allele divergence, the percentage of SNPs per gene length, was 1.9–2.6 for *D. anglica* and “*D. intermedia*” (ID), compared to <0.6 for all other samples (Figure 2; Appendix S1: Table S4). The synthetic hybrids had both locus heterozygosity and allele divergence very similar to, albeit slightly lower than *D. anglica*. The highly elevated nucleotide diversity in *D. anglica* and “*D. intermedia*” (ID) samples and the relatively few long and low‐coverage “paralogs” detected, similar to the synthetic hybrids, further supported that homeologs were too similar to be distinguished and were assembled into chimeric sequences in these datasets. Therefore, next, we used a reference‐based phasing approach in HybPhaser to distinguish between subgenomes.

### Reference‐based phasing showed *D. anglica* and “*D. intermedia”* (ID) subgenomes are highly similar to *D. rotundifolia* and *D. linearis*

**Figure 3:**
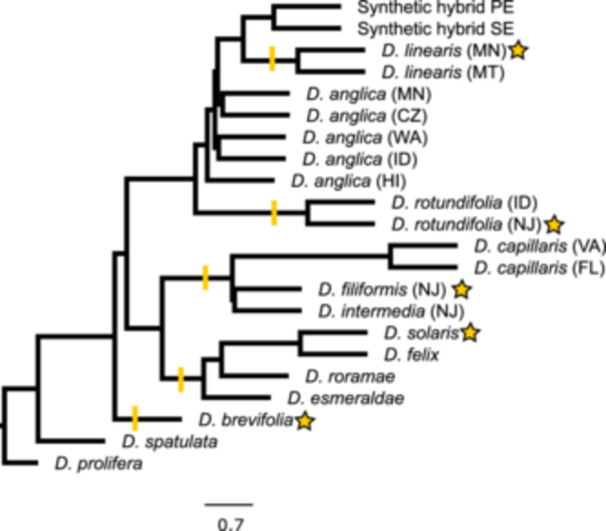
ASTRAL tree estimated from HybPiper assembly without phasing. *Drosera prolifera* was used to root the tree. The scale bar indicates internal branch lengths in coalescent units. All branches have a posterior probability of 1. Terminal branch lengths were artificially chosen. Stars indicate samples used as clade references for phasing, and the yellow lines indicate the clade each clade reference represents.

Based on initial species tree inference, we chose *D. rotundifolia* (NJ), *D. linearis* (MN), *D. solaris* Fleischm., Wistuba & S. McPherson, *D. brevifolia* Pursh, and *D. filiformis* as clade references for phasing in HybPhaser (Figure 3). Despite having a genome size larger than the tetraploid *D. anglica* (Table 2; Appendix S1: Table S1), the 2*n* = 20 chromosome count and low heterozygosity and sequence divergence of *D. filiformis* confirmed that it is a diploid and is suitable as a clade reference for mapping reads.

Results of read mapping to clade references showed that most samples either had >14% of reads unambiguously mapping to a single clade reference or <9% of reads unambiguously mapping to multiple references. In contrast, all *D. anglica* samples, “*D. intermedia*” (ID), and synthetic hybrids each had >16% of reads mapping unambiguously to two clade references: *D. rotundifolia* (NJ) and *D. linearis* (MN; Appendix S1: Table S3). In total, 3057 genes were assembled in all subgenomes and diploid samples with HybPiper. Allele divergence in the phased *D. anglica* and “*D. intermedia*” (ID) samples decreased but remained higher in the *D. linearis* subgenome than the *D. rotundifolia* subgenome of all samples (Figure 2; Appendix S1: Table S4). Similarly, locus heterozygosity remained high for most *D. anglica* and “*D. intermedia*” (ID) samples after phasing, likely due to partial sequences in the reference transcriptome assemblies, despite our requirement that the gene be present in both clade references. In addition, lineage‐specific mutations in the parental and allopolyploid genomes may lead to incomplete phasing. After phasing, *D. anglica*, “*D. intermedia*” (ID), and synthetic hybrids each had 177–223, 239, and 37–123 more genes, respectively, recovered from phasing to *D. linearis* than to *D. rotundifolia* (Appendix S1: Table S4). From here on, the subgenome phased to *D. linearis* will be referred to as subgenome L, and the subgenome phased to *D. rotundifolia* will be referred to as subgenome R.

### Phylogenomic analyses supported the affinity of *D. anglica* with *D. linearis* and *D. rotundifolia*

**Figure 4:**
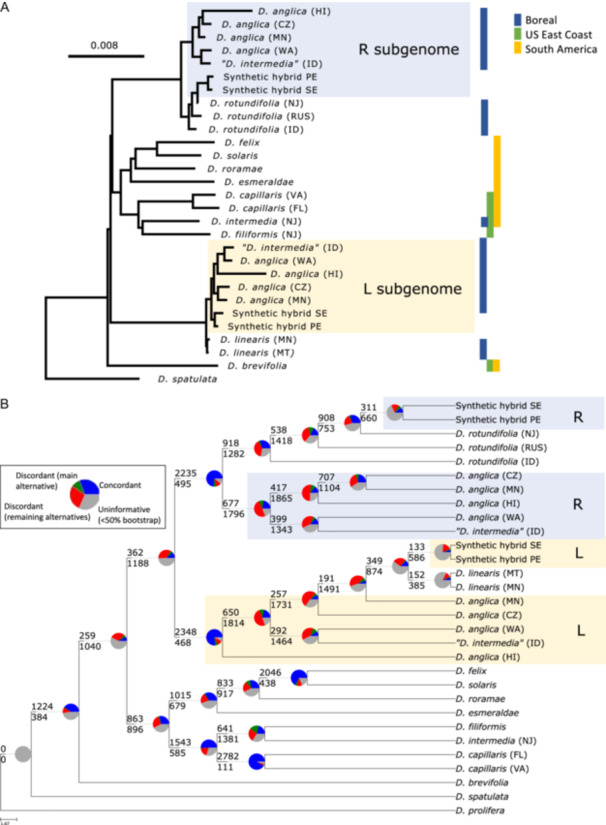
Phylogenies of *Drosera* sect. *Drosera* estimated from 3569 genes assembled using HybPiper with the subgenomes of *D. anglica* phased using HybPhaser. (A) RAxML phylogram. The scale bar indicates branch length. All nodes had 100% bootstrap support except the node uniting *D. linearis* (MT) and *D. linearis* (MN), which had a bootstrap support of 71%. (B) ASTRAL cladogram with node support from PhyParts. *Drosera prolifera* was used to root both the RAxML and ASTRAL trees but is removed from the RAxML tree due to the very long branch length. The full tree can be found in Appendix S5.

All phylogenomic analyses, unphased (Figure 3) or phased (Figure 4; Appendix S5), supported the affinity of *D. anglica* with *D. linearis* and *D. rotundifolia*—none of the remaining species of *D.* sect. *Drosera* were sister to *D. anglica*. Using the 3057 genes assembled in every sample and phased subgenome, phylogenetic analyses in ASTRAL and RAxML both strongly supported each phased subgenome grouping with its respective clade reference (Figure 4; Appendix S5). Both RAxML and ASTRAL recovered sequences from the *D. anglica* R subgenome being monophyletic (677/2473 informative bipartitions) and sister to *D. rotundifolia* + phased subgenomes of the synthetic hybrids. On the other hand, sequences from the L subgenome of *D. anglica* were paraphyletic with *D. linearis* either nested in *D. anglica* (ASTRAL) or monophyletic with *D. linearis* sister to the L subgenome clade (RAxML). Compared to the R subgenome, the L subgenome had shorter branch lengths, less informative bipartitions, and higher levels of gene tree discordance among populations (Figure 4; Appendix S5).

All *D*. sect. *Drosera* species that occurred exclusively in South America (*D. felix* Steyerm. & L.B. Sm., *D. solaris*, *D. esmeraldae* (Steyerm.) Maguire & Wurdack, and *D. roraimae* (Klotzsch ex Diels) Maguire & J.R. Laundon) were monophyletic with strong support (supported by 1015/1694 genes each with >50 bootstrap, referred to as informative from now on; Figure 4). Sister to this clade was a clade of eastern North American species, many reaching South America (*D. filiformis*, *D. intermedia*, and *D. capillaris*; 1543/2128 informative genes; Figure 4). However, species with a broad distribution in both North and South America were not monophyletic (*D. intermedia*, *D. capillaris*, and *D. brevifolia*). Across the section, while some closely related species show similar distributions, neither the boreal nor the longitudinally distributed species were monophyletic.

### The parentage of each *D. anglica* subgenome is further supported by genetic distance

Given the short branch lengths and the lack of phylogenetic signal, we next used pairwise genetic distance to further clarify the relationships among *D. anglica* subgenomes and their parental lineages. Since pairwise genetic distance is sensitive to assembly and alignment error, missing data, and short sequences, we further inspected the multiple sequence alignments after phasing. Visual inspection found that removing alignments with an average pairwise genetic distance <98.0% reduced genes with assembly issues. After removing all gaps in the alignment and alignments shorter than 1000 bp, 140 genes remained. Overall, the median distance between samples ranged from 0 to 0.023, with the highest distance being between the outgroup *D. spatulata* and *D. anglica* (HI; Appendix S6).

The mean distance between *D. rotundifolia* and *D. linearis* samples was 0.016 (Appendix S6). The mean genetic distance between samples of *D. anglica* subgenome L ranged from 0.0005 to 0.0010, with a higher value in *D. anglica* (HI) (0.0020–0.0023; Table 3; Appendix S6). Similarly, the genetic distance between *D. anglica* subgenome L and *D. linearis* ranged from 0.0005 to 0.0020, with the highest values from *D. anglica* (HI) (Table 3; Appendix S6). On the other hand, there was more variation in the *D. anglica* subgenome R. The mean genetic distance between *D. anglica* subgenome R and *D. rotundifolia* ranged from 0.0019 to 0.0040 (Table 3; Appendix S6). The genetic distance between the R subgenomes of *D. anglica* samples ranged from 0.0005 to 0.0028 with the MN‐CZ and “*D. intermedia*” (ID) and *D. anglica* (WA) pairs having a genetic distance of only 0.0005 (Table 3; Appendix S6). The L and R subgenomes were each more closely related to their putative parental lineages than to each other, and the R subgenome had a higher level of genetic diversity than the L subgenome.

**Table 3:** Mean pairwise genetic distance (A) between *D. rotundifolia* and *D. anglica* subgenome R and (B) between *D. linearis* and *D. anglica* subgenome L from 140 genes after phasing. Darker shading indicates greater divergence between samples. Pairwise distances between all samples are available in Appendix S6.

(A) Mean pairwise genetic distance between *D. rotundifolia* and *D. anglica* subgenome R										
Sample	*D. anglica* (HI)	*D. anglica* (CZ)	*D. anglica* (MN)	“*D. intermedia*”(ID)	*D. anglica* (WA)	Synthetic hybrid (SE)	Synthetic hybrid (PE)	*D. rotundifolia* (ID)	*D. rotundifolia* (NJ)	*D. rotundifolia* (RUS)
*D. rotundifolia* (RUS)	0.0038	0.0022	0.0021	0.0021	0.0021	0.0007	0.0008	0.0011	0.0007	0.0000
*D. rotundifolia* (NJ)	0.0039	0.0023	0.0021	0.0021	0.0021	0.0000	0.0001	0.0010	0.0000	
*D. rotundifolia* (ID)	0.0040	0.0024	0.0022	0.0019	0.0020	0.0010	0.0011	0.0000		
Synthetic hybrid (PE)	0.0040	0.0024	0.0022	0.0022	0.0022	0.0001	0.0000			
Synthetic hybrid (SE)	0.0039	0.0023	0.0021	0.0021	0.0021	0.0000				
*D. anglica* (WA)	0.0027	0.0011	0.0010	0.0005	0.0000					
“*D. intermedia*” (ID)	0.0028	0.0012	0.0011	0.0000						
*D. anglica* (MN)	0.0023	0.0005	0.0000							
*D. anglica* (CZ)	0.0021	0.0000								
*D. anglica* (HI)	0.0000									

(B) Mean pairwise genetic distance between *D. linearis* and *D. anglica* subgenome L.									
	*D. anglica* (HI)	*D. anglica* (CZ)	*D. anglica* (MN)	“*D. intermedia*” (ID)	*D. anglica* (WA)	Synthetic hybrid (SE)	Synthetic hybrid (PE)	*D. linearis* (MN)	*D. linearis* (MT)
*D. linearis* (MT)	0.0020	0.0008	0.0006	0.0006	0.0005	0.0000	0.0001	0.0000	0.0000
*D. linearis* (MN)	0.0020	0.0008	0.0006	0.0006	0.0005	0.0000	0.0002	0.0000	
Synthetic hybrid (PE)	0.0021	0.0009	0.0006	0.0007	0.0006	0.0001	0.0000		
Synthetic hybrid (SE)	0.0020	0.0008	0.0006	0.0006	0.0005	0.0000			
*D. anglica* (WA)	0.0020	0.0008	0.0007	0.0005	0.0000				
“*D. intermedia*” (ID)	0.0022	0.0010	0.0008	0.0000					
*D. anglica* (MN)	0.0022	0.0008	0.0000						
*D. anglica* (CZ)	0.0023	0.0000							
*D. anglica* (HI)	0.0000								

### Analyses of *rbcL* sequences with expanded taxon sampling and ribosomal sequences both recovered a close relationship and near‐identical sequences between *D. anglica* and *D. linearis*

**Figure 5:**
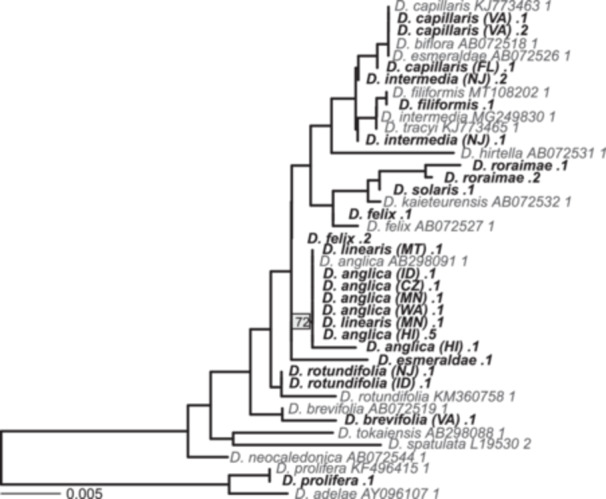
Phylogeny inferred from plastid *rbcL* sequences using RAxML strongly supports the monophyly of *Drosera anglica* + *D. linearis* (bootstrap = 72, see box on node). Newly sequenced samples are bold and black; samples from NCBI GenBank include accession numbers and are gray. Each newly sequenced sample is labeled with a number indicating which assembled sequence it represents. Missing numbers were sequences removed due to length shorter than 300 bp or a tip with a spurious long branch. The scale bar on the lower left indicates branch length. Phylogeny with all the bootstrap values is available in Appendix S7.

From the *rbcL* phylogeny inferred from newly sequenced and previously published Sanger‐sequencing data, all samples of *D*. *linearis* and *D. anglica* formed a well‐supported clade (72% bootstrap; Figure 5; Appendix S7) with *D. rotundifolia* recovered in a separate clade. Additionally, all *D*. *anglica rbcL* sequences are identical to *D. linearis* except for three variants unique to the *D. anglica* (HI) sample.

**Figure 6:**
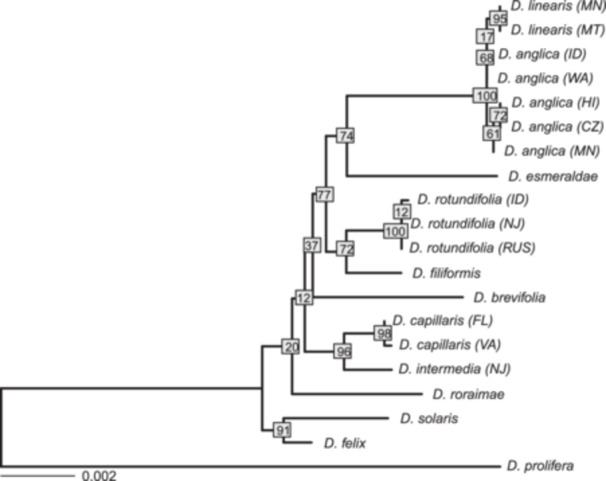
Phylogenetic analysis of rDNA cistron sequences using RAxML strongly supports *Drosera anglica* samples being paraphyletic with *D. linearis* nested among them. Bootstrap support values are labelled on nodes. Scale bar on the lower left indicates branch length.

Similarly, all *D. anglica* samples, whether sequenced with DNA or RNA, show homozygous SNPs predominantly matching *D. linearis* across the nuclear ribosomal region. At 30 SNP locations, all *D. linearis* and *D. anglica* samples were homozygous for one variant and all *D. rotundifolia* samples were homozygous for the other variant (Appendix S8). At only one SNP location, *D. rotundifolia* and *D. anglica* were homozygous for the same allele, while *D. linearis* was homozygous for a different allele (Appendix S8). Due to the secondary structure in the internal spacers of the ribosomal cistron, we were only able to extract SNPs from the newly sequenced samples. Therefore, we were unable to combine the resulting SNP alignment with previously published Sanger sequencing data. Nonetheless, from the phylogenetic analysis of the SNP alignment, we found strong support (100% bootstrap) for the monophyly of all *D. anglica* and *D. linearis* samples (Figure 6).

### *Drosera anglica*'s genome has doubled in size compared to most other diploid North American species

We generated genome size estimates for the Washington and Idaho populations of *D. rotundifolia, D. anglica*, and “*D. intermedia*” (Table 2; Appendix S1: Table S1). Together with previously reported genome sizes (Veleba et al., 2017; Mohn et al., 2023), diploid genome sizes for *Drosera* sect. *Drosera* are highly variable, ranging from 0.6 Gb in *D. spatulata* to 5.9 in *D. tracyi*. North and South American *Drosera* with a chromosome count of 2*n* = 20 had diploid genome sizes mostly between 1.6 Gb and 2.7 Gb except for 4.9 and 5.9 Gb for *D. filiformis* and *D. tracyi*, respectively. Despite their large genome size, *D. filiformis* and *D. tracyi* both have a diploid chromosome count of 2*n* = 20 (Wood, 1955; Hoshi and Kondo, 1998) and exhibit allele divergence and locus heterozygosity similar to those of other diploids. Therefore, their larger genome size was likely due to the proliferation of repetitive elements rather than polyploidy. The genome size of *D. anglica* ranged from 4.6 to 4.7 Gb, approximately twice the size of most of the diploid species, including *D. rotundifolia*. Genome size varied within species, both between studies and among populations, with our newly generated *D. anglica* (WA) being 75 Mb smaller than previous estimations of *D. anglica*, and *D. rotundifolia* (ID) was 400 Mb smaller than previous studies. The “*D. intermedia*” (ID) population had a genome size more than twice that of *D. intermedia* and similar to, though approximately 660 Mb higher than, the *D. anglica* (WA) population. There are no genome size estimates for *D. linearis*.

## DISCUSSION

By comparing transcriptome and genome sequences in multiple populations across the range of *D. anglica*, we found strong evidence for the origin of the allotetraploid *D. anglica* from the diploid lineages represented by *D. linearis* and *D. rotundifolia*. Between the two, *D. linearis* represents the plastid lineage and the dominant nuclear rDNA cistron. Additionally, we confirmed that the disjunct “*D. intermedia*” population in northern Idaho is *D. anglica* with evidence from both sequence and genome size. Comparing *D. anglica* populations with parental lineages, we found no evidence for multiple independent origins of the European, North American, and Hawaiian populations or ongoing gene flow from either parental lineage into *D. anglica*. More broadly, in *D*. sect. *Drosera*, two geographic distributions (boreal and the east coast of North America to South America) are not reciprocally monophyletic, suggesting that species diversification in the section may be associated with multiple range shifts. Similarly, leaf length‐to‐width ratios have shifted at least twice because neither species with narrow, long leaves nor short, round leaves are monophyletic. Finally, our work highlights the value of phasing data to evaluate parental lineages and presents a cautionary tale that visualization of assemblies and alignments is essential in identifying errors and chimeric assemblies and interpreting phylogenomic data.

### *Drosera linearis* and *D. rotundifolia* represent the parental lineages of *D. anglica*

Results from our phylogenetic analyses, identical or near‐identical sequences, geographic distributions, microhabitats, and leaf morphology all support *D. linearis* and *D. rotundifolia* as representing the parental lineages of *D. anglica*. Sequence assembly of *D. anglica* with Trinity or HybPiper alone resulted in chimeric genes due to the high sequence similarity (98.45%) between the subgenomes (Table 3; Appendix S1: Appendix S6). The highly similar subgenomes and chimeric assembly also explain the lack of an *D. anglica‐*specific allopolyploidy peak in the Ks plots. By mapping reads to clade reference transcriptome assemblies using HybPhaser, we found higher similarity of *D. anglica* subgenomes to *D. rotundifolia* and *D. linearis*, respectively, than to *D. brevifolia*, *D. filiformis*, or *D. solaris* as representatives of other *D*. sect. *Drosera* clades (Appendix S2). After phasing the transcripts, we found close phylogenetic affinity (Figure 4; Appendix S5) and near‐identical sequences between *D. anglica* subgenomes and *D. linearis* (99.79–99.95%) and *D. rotundifolia* (99.60–99.80%), respectively (Table 3; Appendix S6).

In addition to the extremely high sequence similarity between *D. anglica* subgenomes and their respective parental lineages, an extant unsampled species representing a closer parental lineage is unlikely, given distribution, habitat, and morphology. Our sampling included all three boreal species, all clades with linear leaves, and all species with which *D. anglica* co‐occurs; most of the unsampled species (7 of 9) are restricted to South America (Figure 4; Appendix S5; Fleischmann and Gonella, 2018). Lastly, both *D. rotundifolia* and *D. linearis* are unique in their morphology and habitat, and no closely related species are known. Given that representative species from each clade were tested and compared as alternative phasing references, we were able to exclude the possibility that the resulting subgenome affinity is an artifact of the choice of phasing references.

Given the identical or near‐identical *rbcL* sequences between *D. anglica* and *D. linearis* (Figure 5; Appendix S5), it is highly likely that the plastid of *D. anglica* originated from *D. linearis* or an extinct close relative. This pattern is unlikely to be an artifact of RNA editing because the *rbcL* sequence recovered from our genome sequencing of the Hawaiian population of *D. anglica* is identical to those recovered from transcriptomic data, and *D. rotundifolia* has reduced RNA editing (Gruzdev et al., 2019). Although plastids are generally maternally inherited in plants (Greiner et al., 2015), plastid DNA has been detected in the pollen of *Drosera muscipula* and *D. capillaris*, suggesting potential for paternal or biparental inheritance of plastids in *Drosera* (Corriveau and Coleman, 1988). Therefore, here we use the term “plastid donor lineage” instead of “maternal lineage”. A previous phylogenetic study proposed that *D. rotundifolia* is the plastid donor of *D. anglica* because their *rbcL* sequences only differed by 3 bp (Rivadavia et al., 2003). However, Rivadavia et al. (2003) did not sample *D. linearis*, and the similarity reflected the relatively slow evolutionary rate of *rbcL* and the close relationship between *D. rotundifolia* and *D. linearis*.

While the nuclear rDNA cistron is initially biparentally inherited, previous work in polyploids has shown that it can undergo rapid homogenization (e.g., Pontes et al., 2004). Interestingly, in *D. anglica*, we only detected the *D. linearis* copy of the rDNA cistron in all five of our *D. anglica* transcriptome and genome sequence datasets (Figure 6; Appendix S8). In other words, a relatively rare species with a narrow distribution (*D. linearis*) contributed the dominant rDNA cistron to a widespread tetraploid. A similar pattern of gene conversion to a single ribosomal copy (Wang et al., 2023) or expression of a single subgenome was observed in both *Gossypium* (Cronn et al., 1999) and *Brassica napus* (Adams et al., 2003). The overall dominance of the L subgenome is also supported by the higher number of genes recovered from the L subgenome in both transcriptome and genome data sets. Higher gene recovery in the L subgenome was also seen, though to a lesser extent, in the synthetic hybrids. This recovery rate could be due to the higher number of reads (although an equal number of base pairs) from *D. linearis* were used to generate the synthetic hybrid, bias during phasing, or differences in completeness and quality of the clade references. Analyses of additional genes in transcriptomic and genomic data with synteny information are needed to quantify the degree to which the L subgenome is dominant in gene expression and gene retention. The inability to detect both parental lineages in rDNA emphasizes the value of sampling additional nuclear loci to identify subgenomes.

Despite the higher level of genetic similarity between *D. linearis* and *D. anglica* subgenome L than between *D. rotundifolia* and *D. anglica* subgenome R (Table 3; Appendix S6), the homeologous chromosomes of *D. anglica* and *D. linearis* do not pair properly in hybrids, unlike the homeologous chromosomes of *D. rotundifolia* and *D. anglica* (Table 1; Kondo and Segawa, 1988; Gervais and Gauthier, 1999). The improper pairing of chromosomes suggests a chromosome rearrangement event has occurred. Still, synteny analysis is needed to confirm whether the mispairing of chromosomes is the result of a genome structural change or is solely due to an epigenetic difference (Herrera et al., 2008; Fu et al., 2012). If there has been a chromosome rearrangement, since *D. anglica* chromosomes also pair properly with the more distantly related *D. intermedia* (Table 1, Figure 4; Kondo and Segawa, 1988) but *D. linearis* chromosomes do not pair properly with *D. rotundifolia* (Wood, 1955), recent chromosome rearrangement events likely occurred in the *D. linearis* lineage post the split from the parental lineage of *D. anglica* (Gervais and Gauthier, 1999). Gervais and Gauthier (1999) expressed concern that hybridization might dilute and ultimately replace *D. linearis*. The *D. linearis*‐specific chromosome rearrangement suggests a potential mechanism that maintains species boundaries between *D. linearis* and the other co‐occurring *Drosera* species.

### The northern Idaho population previously referred to as *D. intermedia* is *D. anglica*

All evidence, including genome size (Table 2), percentage of heterozygous loci and allele divergence (Figure 2; Appendix S1: Table S4), and phylogenetic analyses based on both reference‐guided and de novo assemblies (Figures 3, 4), indicates that the Idaho population of “*D. intermedia*”, a critically imperiled species in Idaho (Idaho Fish and Game, 2024a), is indeed *D. anglica*, a species not ranked in Idaho (Idaho Fish and Game, 2024b). The diploid genome size of “*D. intermedia*” (ID) was about twice that of *D. intermedia* or *D. rotundifolia* (Table 2; Appendix S1: Table S1). The “*D. intermedia*” (ID) sample had locus heterozygosity and allele divergence similar to that in the known *D. anglica* populations and much higher than in the diploid *D. intermedia* (Figure 2; Appendix S1: Table S4). When mapped to clade representatives, like *D. anglica* and unlike *D. intermedia*, it had a strong affinity to both *D. linearis* and *D. rotundifolia* (Appendix S1: Table S3). In phylogenetic analyses with phased data (Figure 4; Appendix S5), “*D. intermedia*” (ID) was nested among the *D. anglica* samples, confirming that this population belongs to *D. anglica*. From now on, we will refer to it as *D. anglica* (ID).

Despite a strong sequence affinity to *D. anglica*, the genome size of the Idaho population of *D. anglica* was 660 Mb and more than 10% larger than *D. anglica* (WA; Table 2; Appendix S1: Table S1), greater than the variation observed between fresh and silica‐dried samples (Bainard et al., 2011; Wang and Yang, 2016) or between *D. anglica* (WA) and previous measurements of *D. anglica* (75 Mb; Veleba et al., 2017). While newly formed, triploid hybrids between *D. anglica* and *D. rotundifolia* or other species are known to occur in wild populations (Mellichamp, 2016), a triploid individual would result in a smaller instead of a slightly larger genome, and a hexaploid would exhibit higher allele divergence and locus heterozygosity. Additionally, the *D. anglica* (ID) genome lacked any evidence of increased similarity to either parent (Figure 4), as might be the case if it had a separate origin or was backcrossed with a parent. Instead, the slightly larger genome in *D. anglica* (ID) may be due to additional repetitive regions or aneupolyploidy.

Confirming this northern Idaho population as *D. anglica* with molecular and genome size data supports Rice's proposal (2019) based on morphology and habitat. The leaf shape of the *D. anglica* (ID) population falls at the smaller end of the length to width ratio distribution of *D. anglica* (oblong to linear‐spatulate; Mellichamp, 2016; Lowrie et al., 2017) and so is more similar to *D. intermedia*. However, *D. anglica* leaves are erect, although old leaves may become recumbent (Figure 1). In contrast, in mature *D. intermedia* plants, the leaves radiate spherically outward, with lower leaves recurved when the plant has a stem (Figure 1). *Drosera anglica* has an erect peduncle originating vertically from the plant, while *D. intermedia* has a peduncle that emerges close to horizontally before ascending (Figure 1). One potential explanation for the *D. anglica* (ID) population having shorter leaf blades than typical *D. anglica* is its habitat differences from other nearby *D. anglica* populations. While *D. anglica* populations in the region occur on the lakeside edges of floating bogs among *Sphagnum* and buckbean (*Menyanthes trifoliata*) and on floating logs, this population occurs on a sloped fen. Similar leaf‐shape plasticity corresponding to habitat differences has been observed in *D. intermedia* (Banaś et al., 2024).

The populations in northern Idaho and another in south‐central Idaho are over 1000 km west of the nearest *D. intermedia* populations, which has made them a conservation priority in Idaho. With this knowledge of the northern Idaho population being *D. anglica*, the southern Idaho population is also likely *D. anglica* (Rice, 2019). Our results suggest that the conservation status of these populations should be re‐evaluated. More broadly, we observed that *D. intermedia* is often confused with *D. anglica* in herbaria. Our study suggests that additional misidentifications, especially the multiple apparent disjunct populations of *D. intermedia* elsewhere in the world (Figure 1), may be an artifact of misidentification due to phenotypic plasticity.

### Hawaiian *D. anglica* originated by long‐distance dispersal from the boreal region

The Hawaiian sample of *D. anglica* was deeply nested among *D. anglica* from North America and Europe in the phased data (Figure 4; Appendix S5) and showed affinity with the remaining *D. anglica* samples in unphased data (Figure 3). This Hawaiian *D. anglica* sample had higher allele divergences and terminal branch lengths than the remaining *D. anglica* samples which may be partially due to the use of DNA for the Hawaiian *D. anglica* sample instead of RNA. We are likely to recover a more balanced and complete representation of alleles and homeologs from genomic than from transcriptomic sequencing. To ensure that the higher sequence divergence was not an artifact from intron or chimeric assemblies from stitching together exons, before and after phasing we also reanalyzed the *D. anglica* (HI) genome sequence data without stitching contigs in HybPiper, but the result was only a slightly decreased allele divergence. The higher genetic diversity found in the Hawaiian sample is unlikely to be due to a higher ploidy level, as a previous study found a chromosome count of 2*n* = 40 for a Hawaiian population of *D. anglica* (Hoshi and Kondo, 1998). As *D. anglica* is native to only the island of Kaua'i and restricted to the montane wetlands on the island (Mellichamp, 2016), the chromosome count likely came from the same population as or a nearby population to our DNA sample. More likely, the higher genetic diversity and longer terminal branch lengths can also be attributed to a longer growth season, resulting from the loss of winter dormancy on tropical islands (Mellichamp, 2016). Our results add *D. anglica* to the list of Hawaiian plants of boreal origins, such as *Viola* (Violaceae; Marcussen et al., 2012) and *Vaccinium* (Ericaceae; Powell and Kron, 2002).

### A lack of evidence supporting multiple origins of *D. anglica*

Natural hybrids and suspected neopolyploids of *D. linearis* and *D. rotundifolia* have been observed (Wood, 1955), raising the question of whether new allopolyploid hybrids are contributing to the evolution of *D. anglica*. Despite sampling *D. rotundifolia* (ID) 18 and 42 km from *D. anglica* (WA) and *D. anglica* (ID), respectively, and European *D. anglica* and *D. rotundifolia*, the *D. rotundifolia* samples were more closely related to each other than to any *D. anglica* samples (Figure 4; Appendix S5) and the R subgenome of the in silico hybrid was sister to *D. rotundifolia* (NJ), the source sequence of the in‐silico hybrid. Similarly, we observed *D. linearis* being monophyletic but with higher phylogenetic uncertainty due to low sequence divergence (0.1–0.4%, i.e., at most a few base‐pair differences per gene; Table 3; gray in Figure 4B; Appendices S5, S6).

Molecular markers with higher evolutionary rates may be more informative for further testing multiple origins. The higher genetic diversity in the R subgenome among *D. anglica* populations may still come from multiple origins, larger effective population sizes in the ancestral *D. rotundifolia* compared to the *D. linearis* populations, or higher levels of purifying selection in the L subgenome, given its genomic dominance.

The larger population size and widespread distribution of *Drosera rotundifolia* throughout the Pleistocene have been supported by multiple lines of evidence. Pleistocene fossils of *D. rotundifolia* have been found in Canada (Penhallow, 1890, 1896). In addition, Korean populations of *D. rotundifolia* showed low within‐population diversity but high between‐population divergence, suggestive of microrefugia during the last glacial maximum (Chung et al., 2013). On the other hand, *D. linearis* is more restricted in its geography and habitat, and the flarks where it occurs expand and contract more rapidly with climate fluctuations (Kolari et al., 2022). This difference in history may explain the lower genetic diversity in *D. linearis* than in *D. rotundifolia. Drosera anglica*, the allopolyploid hybrid, occurs in an intermediate habitat between bogs and fen flarks that is more abundant than the flarks of *D. linearis* and likely experienced less fluctuation of population sizes than *D. linearis*.

### *Drosera anglica* is intermediate between both parental lineages in most aspects of leaf morphology and microhabitat

The leaf shape and angle of the petiole distinguish *D. anglica* from its parental lineages (Figure 1). The leaf blade of *D. rotundifolia* is wider than long, and the leaves are generally prostrate, spreading, to slightly raised. *Drosera anglica* and *D. linearis* leaves are generally erect, although old leaves may become recumbent. The leaf blades of *D. linearis* are linear, as its name suggests, with the two sides of the leaf parallel and the apex obtuse to truncate. The leaf shape of *D. anglica* is quite variable, ranging from oblong to linear‐spatulate (Mellichamp, 2016; Lowrie et al., 2017). The differences in leaf shape and orientation among *D. rotundifolia*, *D. anglica*, and *D. linearis* correspond to their different microhabitats. At the two extremes, *D. rotundifolia* occurs in drier conditions on sphagnum hummocks, and *D. linearis* occurs in the standing water of fen flarks, naturally occurring swales on a patterned peatland. *Drosera anglica* is often standing in water on the edges and ridges of peatlands and on the edges of floating bogs (Banaś et al., 2023).

Therefore, the oblong to linear‐spatulate leaf shape and the wet peatland edge habitat of *D. anglica* fit the expectation of intermediacy in allopolyploids as *D. rotundifolia* has round leaves and prefers a drier hummock habitat, while *D. linearis* has linear leaves and prefers a standing in mineral‐rich fens swales (Figure 1; Mellichamp, 2016; Lowrie et al., 2017; Banaś et al., 2023). However, some floral features of *D. anglica* differ from those of *D. linearis* and *D. rotundifolia* (Murza and Davis, 2003). For example, *D. anglica* has the smallest and fewest papillate cells on the anther apices of the three species (Murza and Davis, 2003). Cases of features differing from both parents have been previously observed in allopolyploids, likely due to the new genomic context of the genes (Marchant et al., 2016).

When *Drosera* species co‐occur, putative hybrids are occasionally observed with intermediate leaf shapes and habitats (Wood, 1955; Gervais and Gauthier, 1999; Lowrie et al., 2017). For example, in northern Minnesota, we found a plant growing with *D. rotundifolia* on a dry hummock with *D. linearis* nearby. While the habitat suggested *D. rotundifolia*, it had spatulate‐shaped leaves that were more upright in orientation. It had a genome size similar to that of *D. rotundifolia*. This plant produced well‐filled seeds, but we were unable to germinate a small batch of eight.

We note that although leaf shape and position are generally reliable in distinguishing *D. anglica* vs. close relatives, each type of leaf shape is nonmonophyletic across *D*. sect. *Drosera*. Neither long skinny leaves (*D. filiformis*, *D. linearis*) nor round leaves (*D. rotundifolia*, *D. brevifolia*, *D. capillaris*) and neither erect leaves (*D. filiformis*, *D. linearis*, *D. intermedia*, *D. roraimae*) nor prostrate leaves (*D. brevifolia*, *D. capillaris, D. esmeraldae*, and often *D. rotundifolia*) are monophyletic (Figure 4; Mellichamp, 2016; Lowrie et al., 2017). Leaf shape and position are important for prey capture as together they position the leaf blade with sticky traps above water or surrounding plants (Fleischmann et al., 2018).

### *Drosera* sect. *Drosera* has undergone multiple shifts in range

Within *D*. sect. *Drosera*, the two major distributions—boreal, and eastern North America to South America—are nonmonophyletic, indicating that range shifts have occurred multiple times (Figure 4). Of the 15 sections of *Drosera*, *D*. sect. *Drosera* occupies the broadest range in climate and geography. The broad climatic adaptation and geographic distribution are likely achieved via repeated shifts in strategies in prey capture and adaptations to macro‐ and microhabitats.

### Data visualization at multiple stages is important in analyzing large datasets

Our work presents a cautionary tale highlighting the importance of data visualization in multiple steps of data analysis to identify unexpected issues. These issues included sequence processing errors, violations of assembly assumptions, and low‐quality annotations of reference genomes.

Visualizing read mapping in Integrated Genome Viewer (v. 2.12.3; Robinson et al., 2011, 2017) resulted in the detection of a sequence processing error. After cleaning reads and assembling genes, we observed that all single‐end read samples from the same sequencing batch had an increased number of variants on the 3′ end. In addition to residual adaptors, we observed a single T on the 3′ end of many reads. When enough reads with a terminal T ended at the same place, an SNP was called erroneously resulting in overestimating divergence.

HybPiper was designed to assemble genomic reads to coding sequence targets. Therefore, when ends of reads do not map to the target, they are assumed to be introns and are trimmed. When we adopted HybPiper to assemble transcriptomic reads against coding sequence targets from the *D. spatulata* genome annotations, we observed assemblies that had even up to orders of magnitude differences in coverage across the gene with truncated reads, and no reads bridged two adjacent regions. These issues are likely due to either alternative splicing or the presence of chimeric sequences in the reference. To minimize the risk of chimeric sequences, we performed the phylogenomic analysis on single contigs instead of supercontigs for each gene in our transcriptomic data sets.

By visualizing our assemblies, we identified issues with read processing errors in three samples, chimeras in the reference transcripts from a genome assembly, and problems with applying a target enrichment pipeline to transcriptomic data. We were then able to take corrective measures. However, these issues could be easily overlooked without the visualization of assemblies and alignments and could have propagated in our results by overestimating the divergence of sequences.

## CONCLUSIONS

Both reference‐guided and de novo‐based read assembly methods supported *D. rotundifolia* and *D. linearis* as the parental lineages of *D. anglica* with the *D. linearis* lineage being the plastid donor and contributing the dominant subgenome. We found no evidence for multiple origins of *D. anglica*. The Hawaiian *D. anglica* originated through long‐distance dispersal from the boreal region. Future work should further explore subgenomic dominance and population structure in *D. anglica*, including *D. anglica* from Hawaii, and evaluate the presence of a chromosomal rearrangement in *D. linearis*.

Our work highlighted the importance of careful examination of phylogenomic data, including testing for cross‐contamination, and visual inspection of read mapping, SNP patterns, alignments, and individual gene trees. Simply running through standard phylogenomic pipelines would likely omit cryptic reticulation events that are of ecological, evolutionary, and conservation importance.

## AUTHOR CONTRIBUTIONS

R.M. designed the study and led sample collection, lab work, analysis, and writing. Y.Y. assisted with study design, sample collection, analytical approaches, and writing.

## Supporting information


**Appendix S1.** Tables S1–S4. Information for genome size estimation and newly sequenced samples.

**Appendix S2.** Figures S1–S6. Photographic vouchers for samples without an herbarium voucher or with additional features that are not present in herbarium specimens.

**Appendix S3.** The modified PureLink RNA extraction and DNase protocols.

**Appendix S4.** Figures S1, S2. Ks plots showing Ks values.

**Appendix S5.** RAxML tree including *D. prolifera*.

**Appendix S6.** Tables S1, S2. Pairwise genetic distances between all samples from 140 genes ≥1000 bp and ambiguous base pairs removed.

**Appendix S7.** Alignment of *rbcL* variant sites among *D. rotundifolia*, *D. linearis*, and *D. anglica* and the RAxML tree with bootstrap supports.

**Appendix S8.** Alignment of variable sites in rDNA among *D*. *rotundifolia*, *D*. *linearis*, and *D*. *anglica*.

## Data Availability

Raw transcriptomic and genomic reads for newly generated samples are available through the NCBI SRA PRJNA1198097. Code used for analysis, assembled sequences, multiple sequence alignments, and trees are available through Dryad (https://doi.org/10.5061/dryad.7m0cfxq5j).
