# Supplementary material for: Origin of subgenomes in the circumboreal, allopolyploid, carnivorous plant Drosera anglica
Source: Am J Bot. 2026 Mar 2;113(3):e70170. doi: 10.1002/ajb2.70170 (PMC13003725; doi:10.1002/ajb2.70170)
Supplement: Supplementary file 2 — Appendix S2. Figures S1–S6. Photographic vouchers for samples without an herbarium voucher or with additional features that are not present in herbarium specimens. [file AJB2-113-e70170-s007.docx]

**Appendix S2.** Photo vouchers for samples without an herbarium voucher or with additional features not present in herbarium specimens.


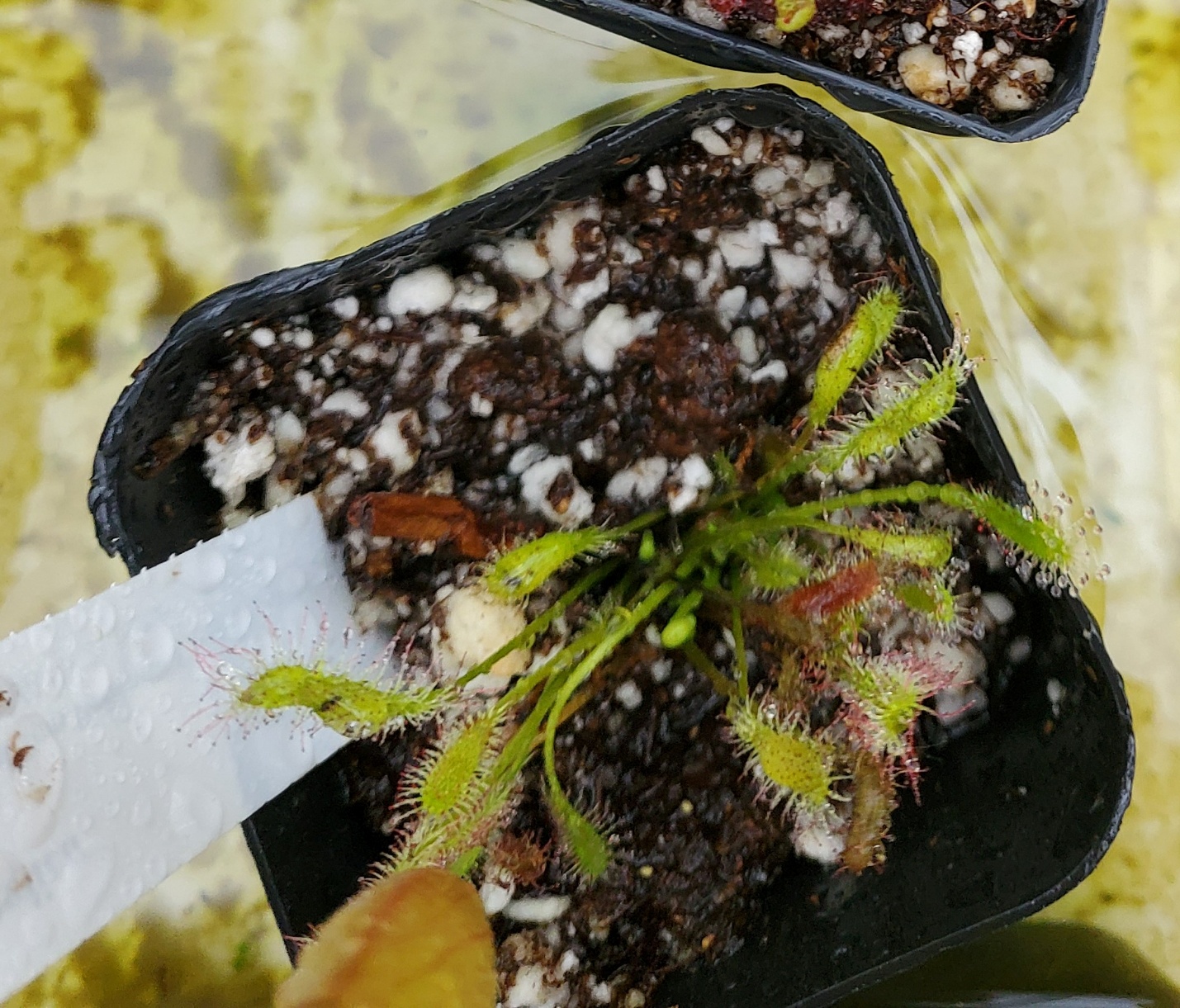


**Figure S1.** *Drosera anglica* (CZ; RM298; cultivated) from Best Carnivorous Plants (BCP ID# D1594, D1614). Locality: Sumava Mts, Southern Bohemia, Czech Republic (CZ).


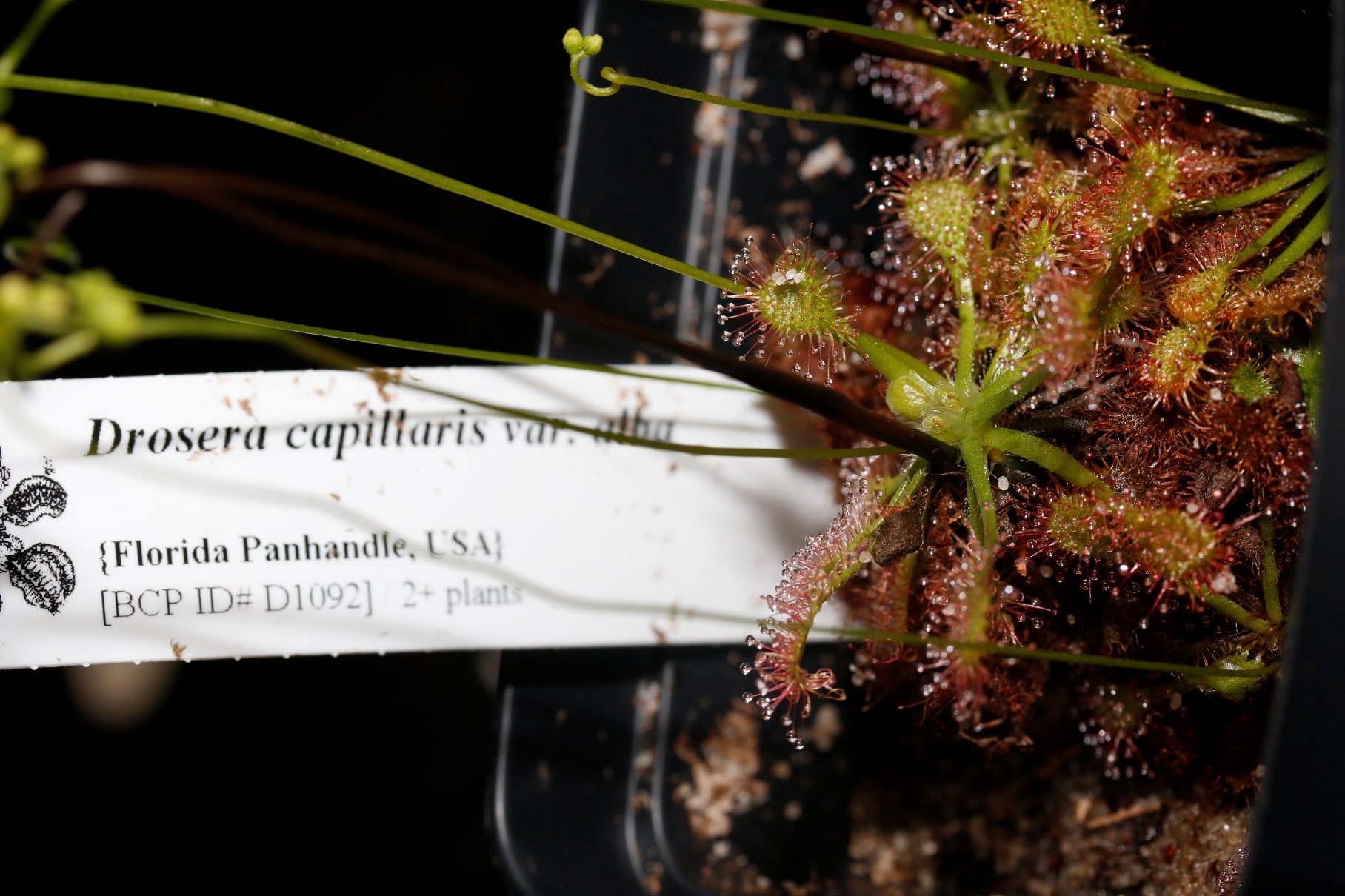


**Figure S2.** *Drosera capillaris* (FL; RM240; cultivated) from Best Carnivorous Plants (BCP ID# D1092). Locality: Florida Panhandle (FL).


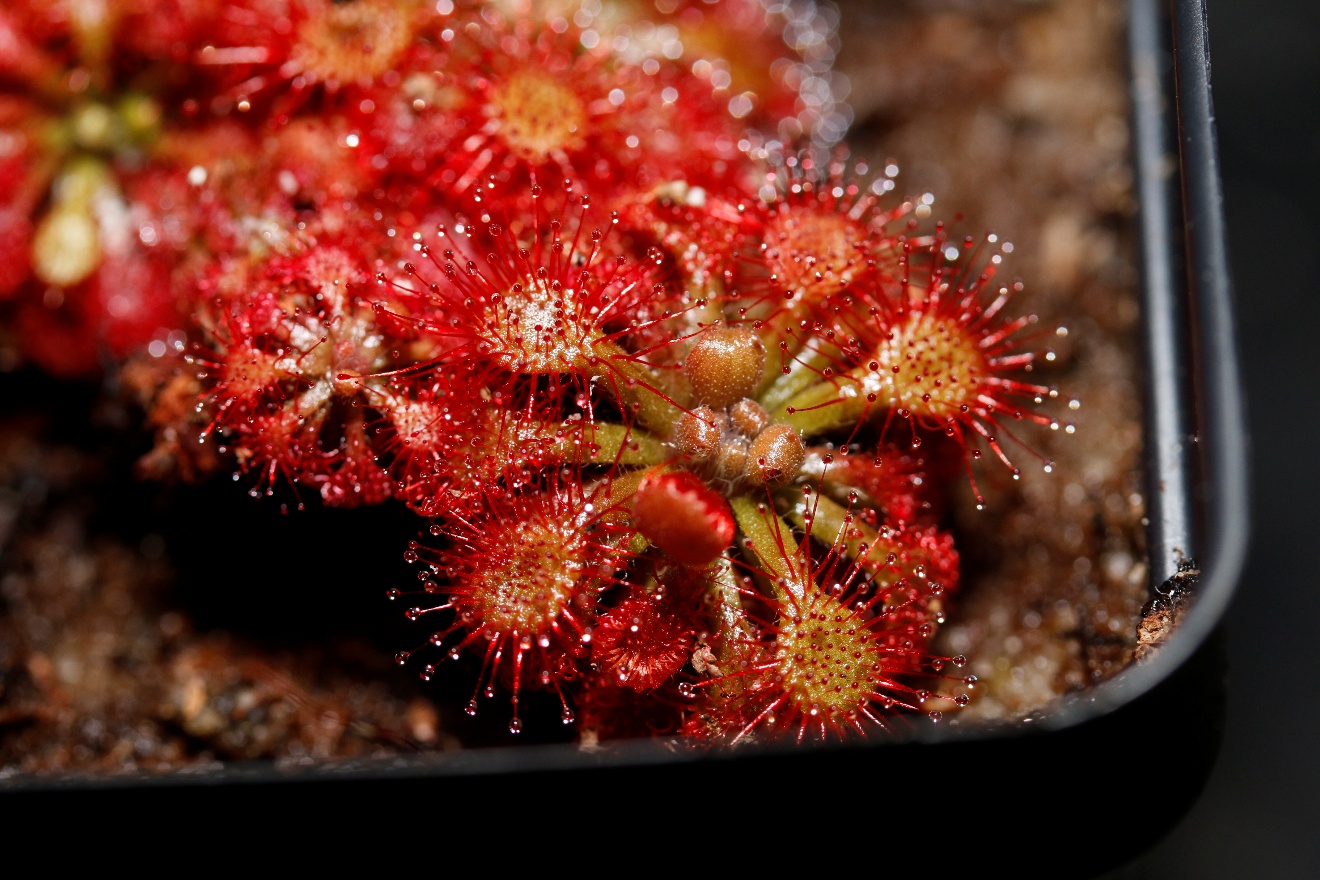


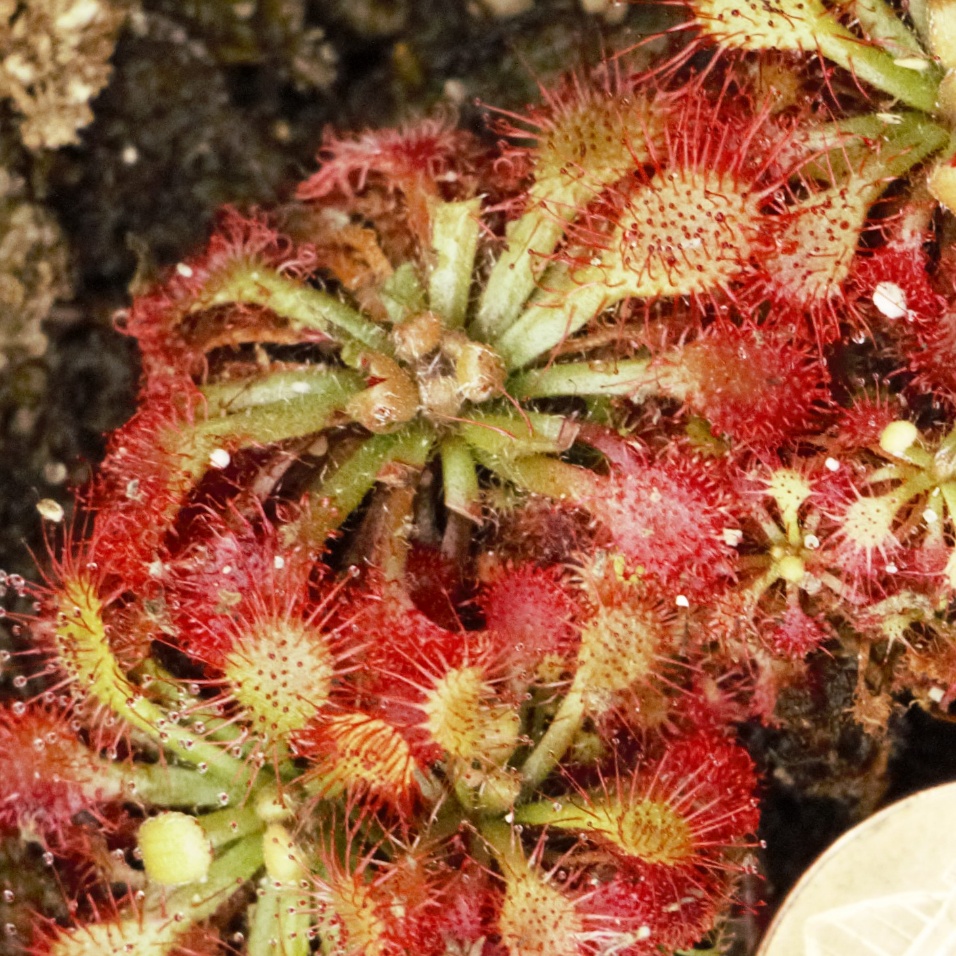


**Figure S3.** *Drosera esmeraldae* (RM241; cultivated) from Best Carnivorous Plants (BCP ID# D761). Locality: Cerro Duida, Venezuela.


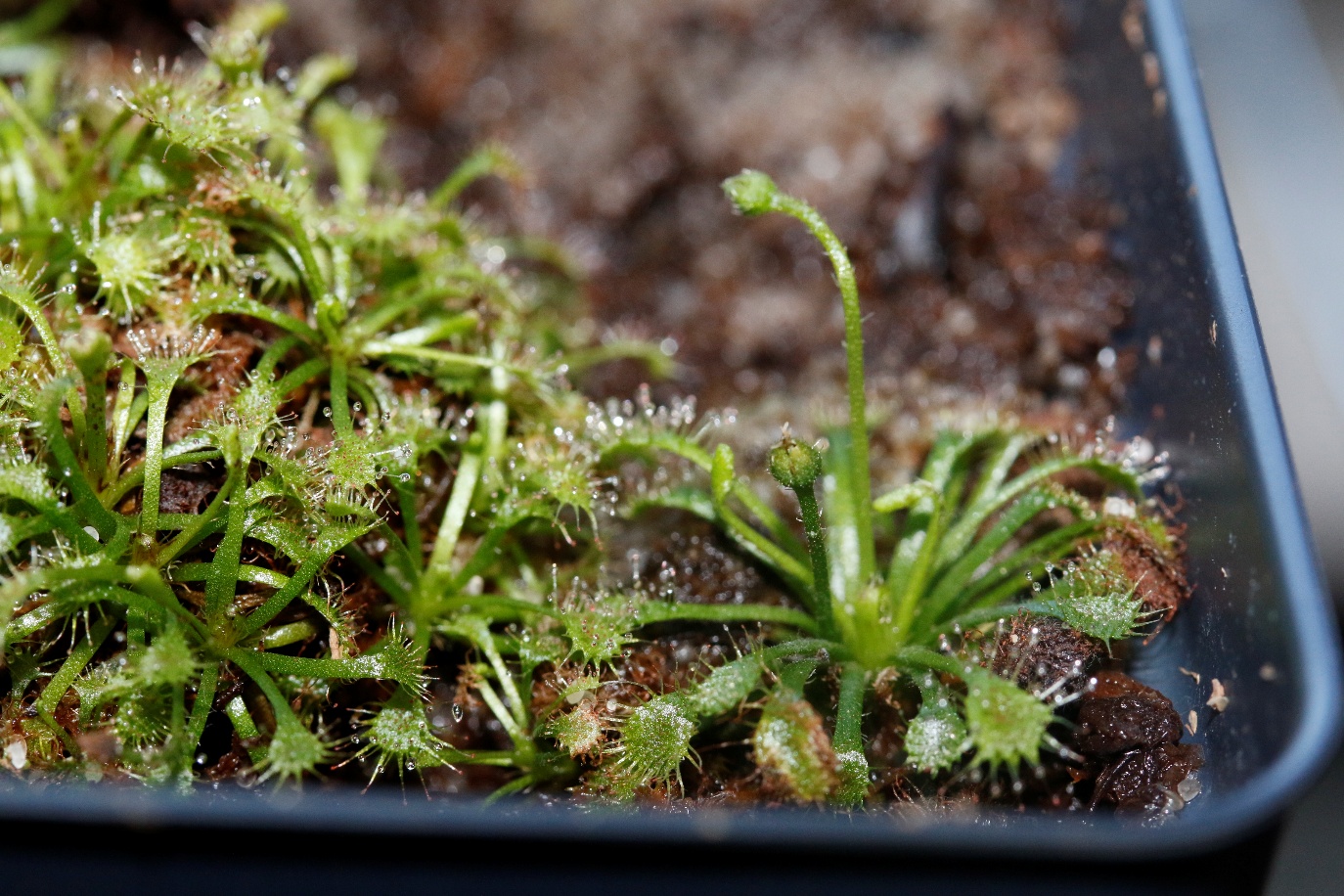


**Figure S4.** *Drosera felix* (RM245; cultivated) from Best Carnivorous Plants (BCP ID# D684, D923, D1834). Locality: Tuku Muruku, Gran Sabana, Brazil.


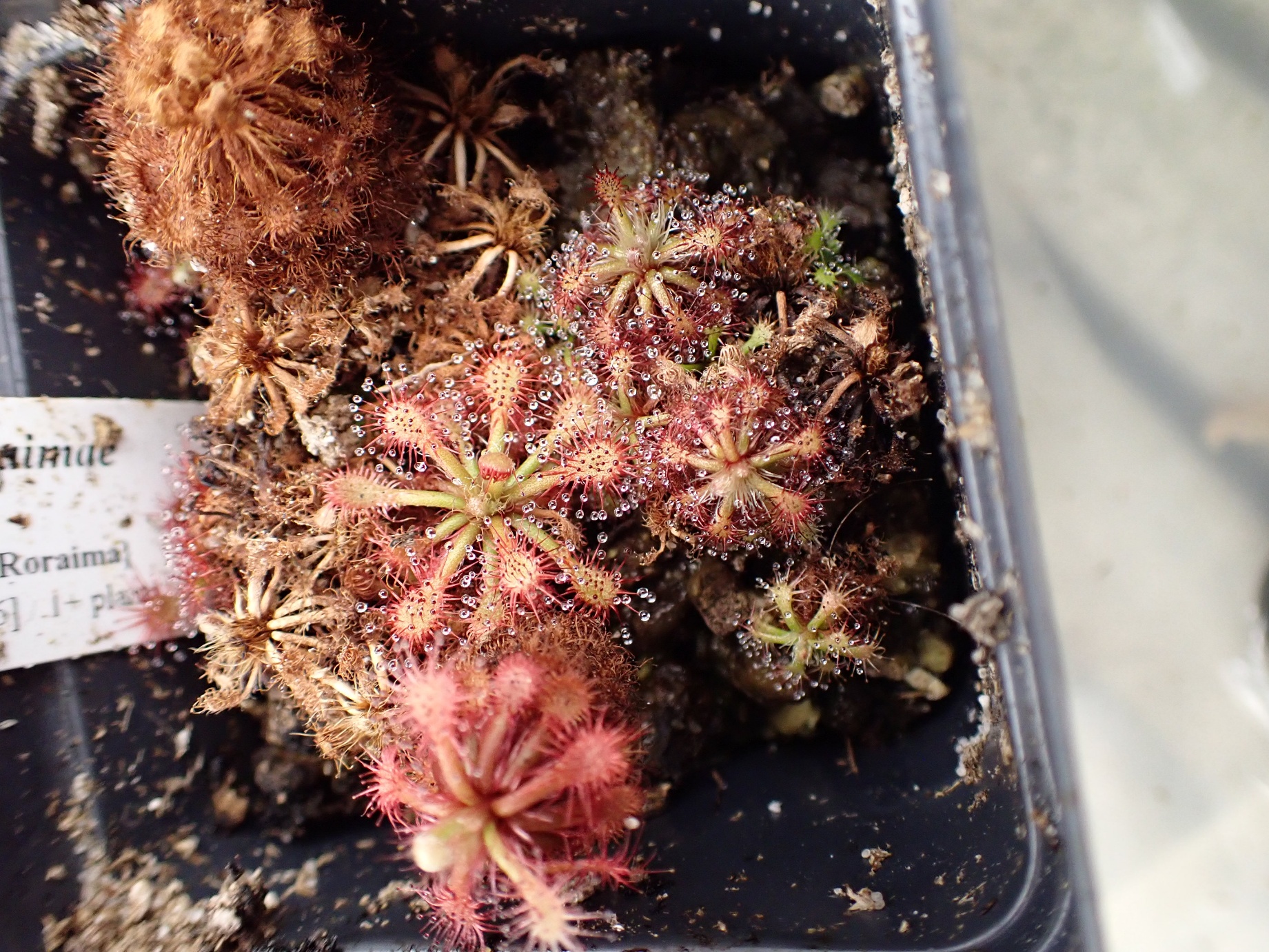


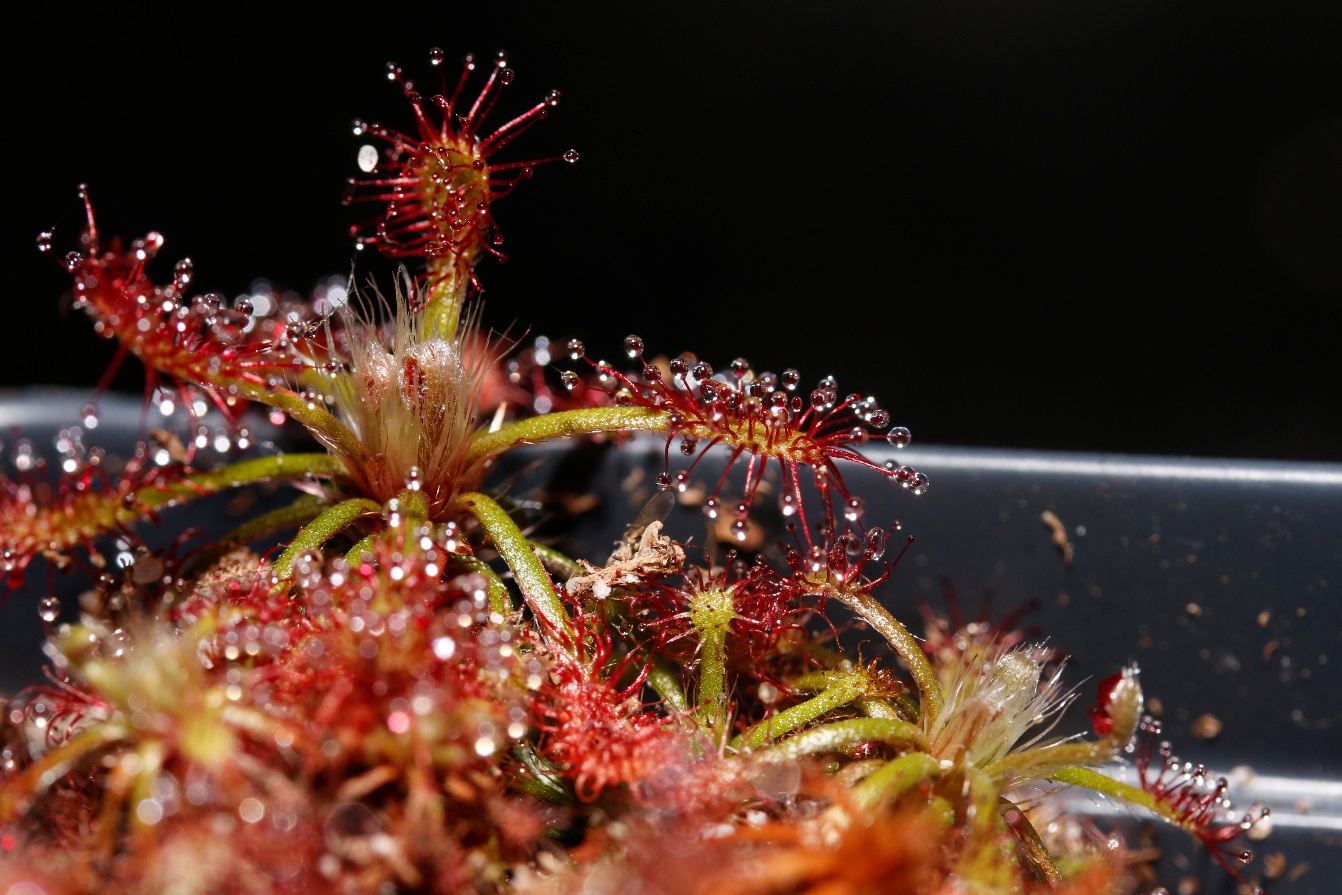


**Figure S5.** *Drosera roraimae* (RM242; cultivated) from Best Carnivorous Plants (BCP ID# D1026). Locality: Summit of Mt. Roraima (which borders Brazil, Guyana, Venezuela).


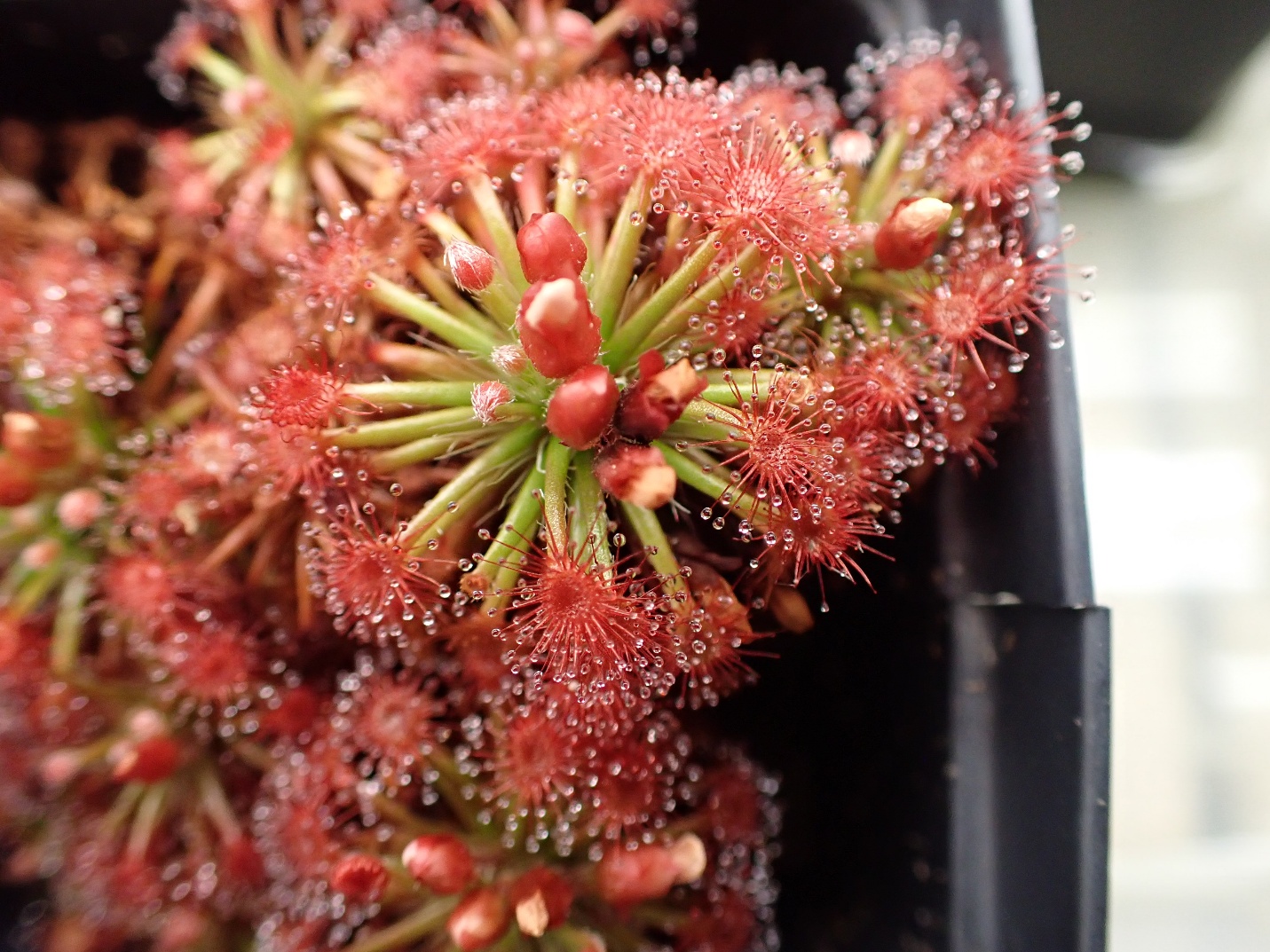


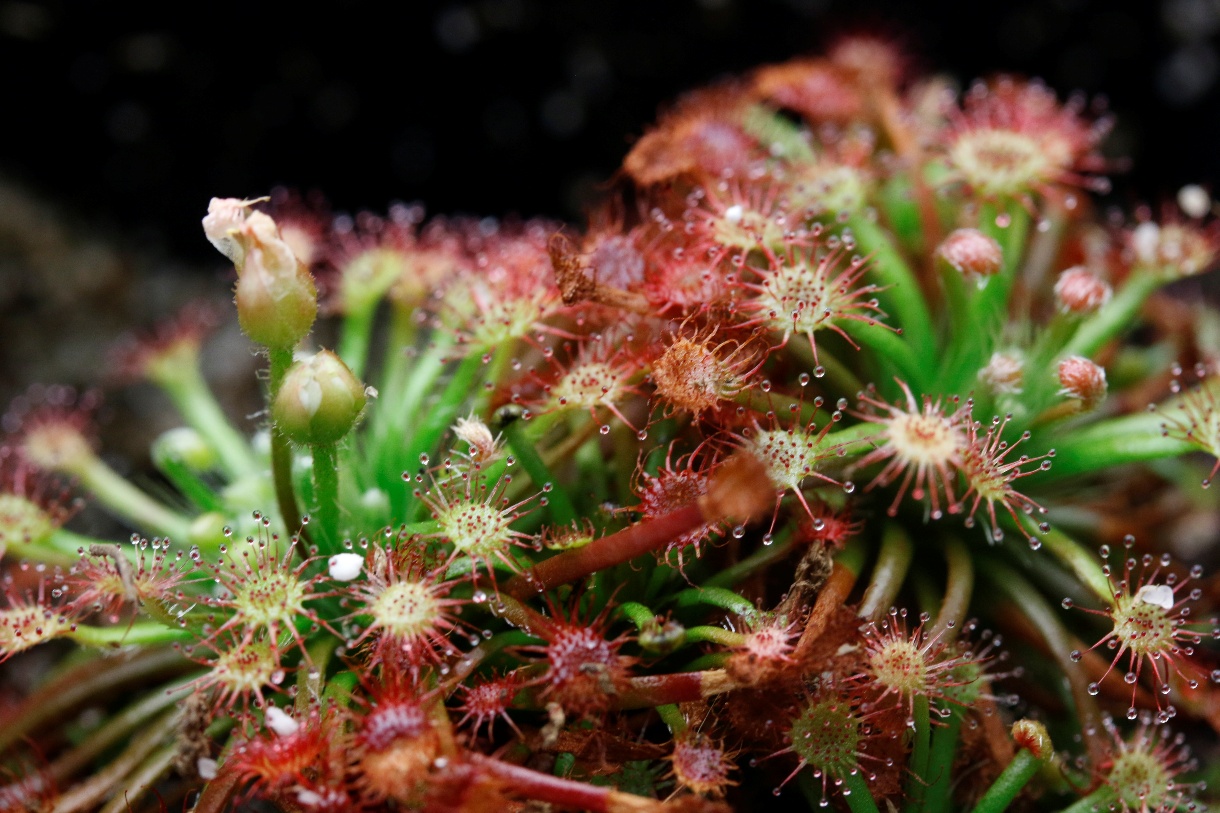


**Figure S6.** *Drosera solaris* (RM237; cultivated) from Best Carnivorous Plants (BCP ID# D982). Locality: Mt Yakontipu, Pakaraima Mountains, Guyana.
