# Supplementary material for: Origin of subgenomes in the circumboreal, allopolyploid, carnivorous plant Drosera anglica
Source: Am J Bot. 2026 Mar 2;113(3):e70170. doi: 10.1002/ajb2.70170 (PMC13003725; doi:10.1002/ajb2.70170)

Appendix S4, Figure S1: Ks plots showing Ks values between 0 and 0.5.

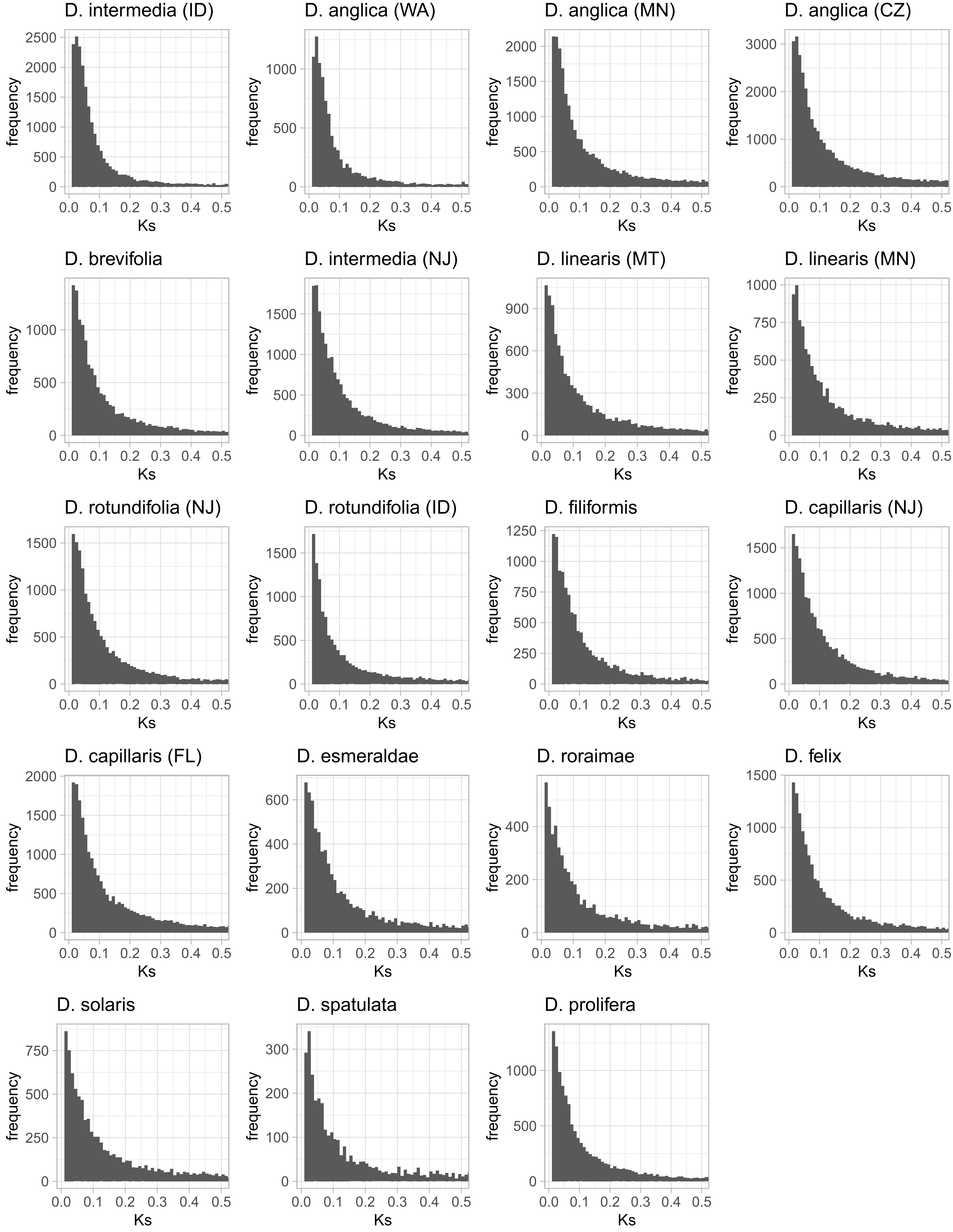

Appendix S4, Figure S2: Ks plots showing Ks values between 0 and 2.5.

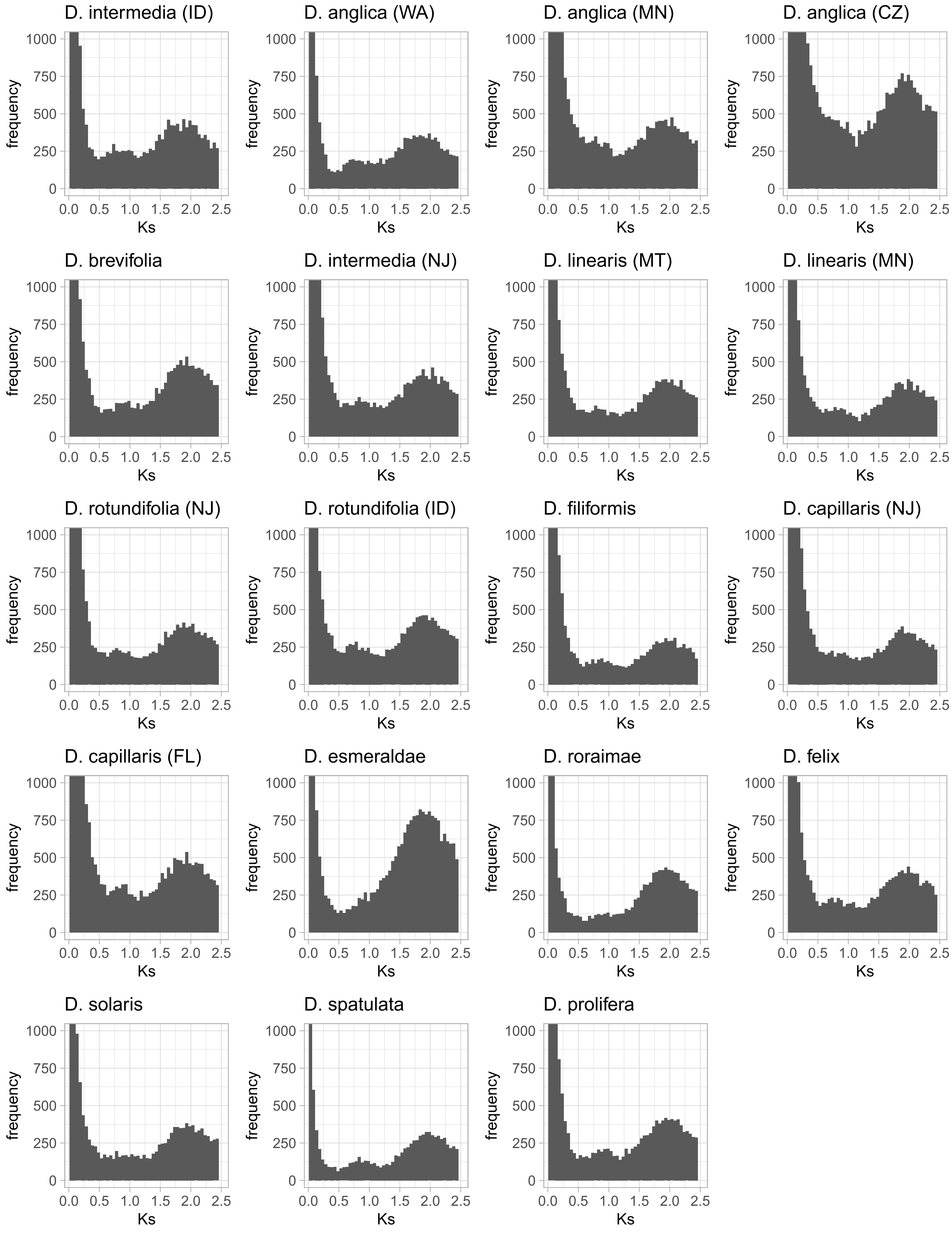

Supplement: Supplementary file 4 — Appendix S4. Figures S1, S2. Ks plots showing Ks values. [file AJB2-113-e70170-s006.pdf]
