# Supplementary figures and images for: Origin of subgenomes in the circumboreal, allopolyploid, carnivorous plant Drosera anglica
Source: Am J Bot. 2026 Mar 2;113(3):e70170. doi: 10.1002/ajb2.70170 (PMC13003725; doi:10.1002/ajb2.70170)

Appendix S5: RAxML tree including *Drosera prolifera*.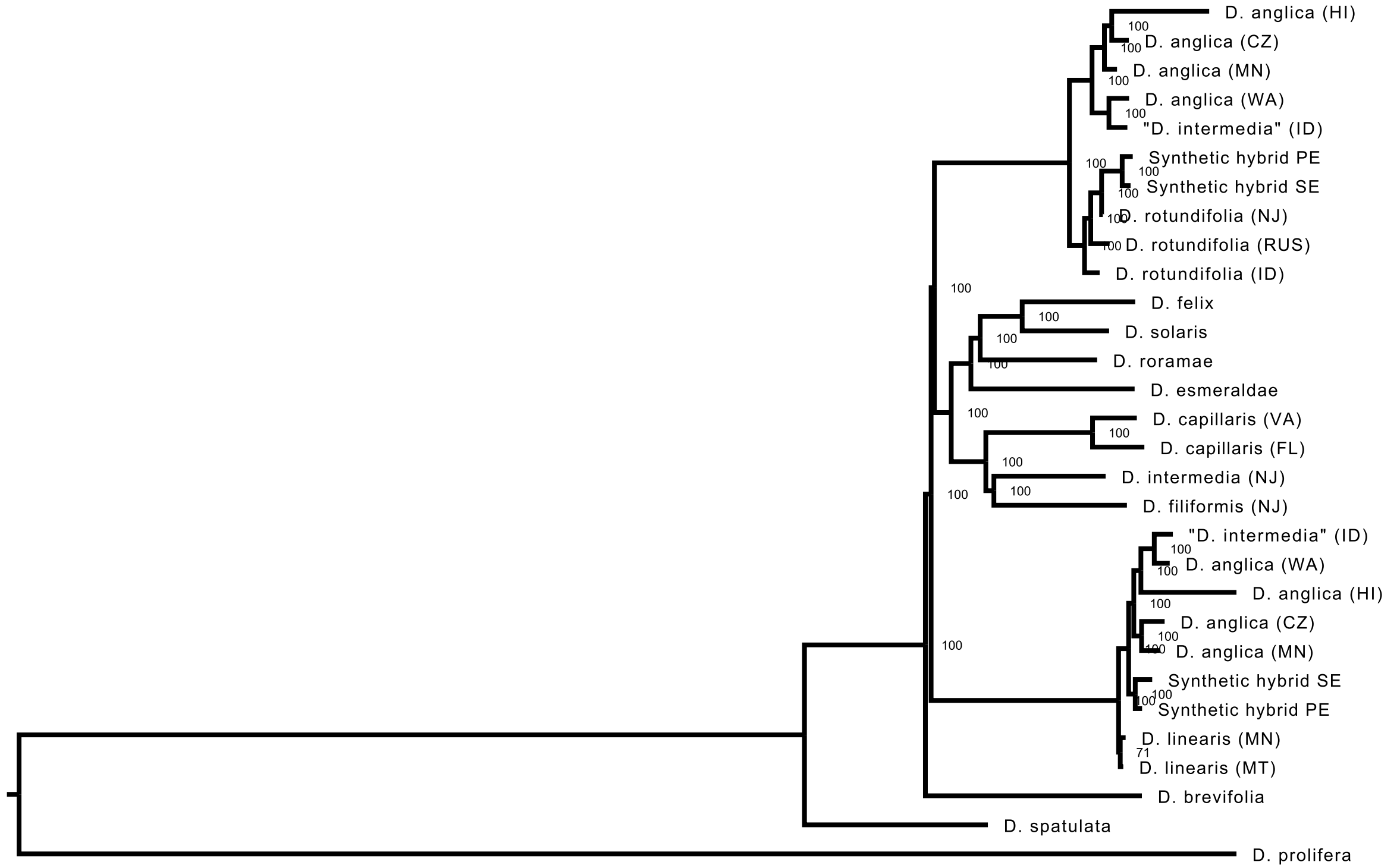

Supplement: Supplementary file 5 — Appendix S5. RAxML tree including D. prolifera. [file AJB2-113-e70170-s001.pdf]
