# Supplementary material for: Origin of subgenomes in the circumboreal, allopolyploid, carnivorous plant Drosera anglica
Source: Am J Bot. 2026 Mar 2;113(3):e70170. doi: 10.1002/ajb2.70170 (PMC13003725; doi:10.1002/ajb2.70170)

Appendix S8. Alignment of variable sites in rDNA among *Drosera rotundifolia*, *D. linearis*, and *D. anglica*. The complete alignment is available in the Dryad repository.

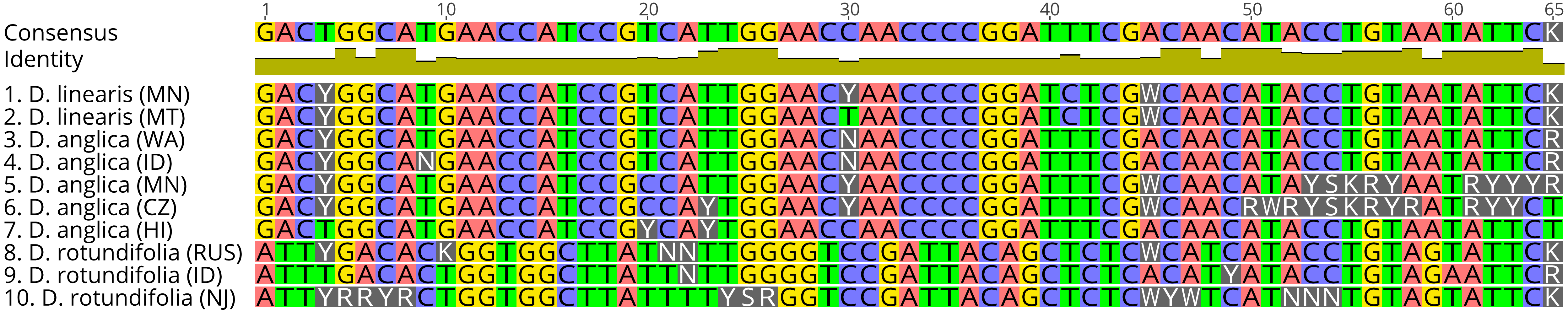

Supplement: Supplementary file 8 — Appendix S8. Alignment of variable sites in rDNA among D. rotundifolia, D. linearis, and D. anglica. [file AJB2-113-e70170-s004.pdf]
